# Effectiveness of engagement initiatives across engagement platforms: A meta-analysis

**DOI:** 10.1007/s11747-023-00925-7

**Published:** 2023-02-28

**Authors:** Markus Blut, Viktorija Kulikovskaja, Marco Hubert, Christian Brock, Dhruv Grewal

**Affiliations:** 1 Durham University Business School, Durham University, Mill Hill Lane, DH1 3LB Durham, UK; 2 AU BSS, Aarhus University, Fuglesangs Allé 4, 8210 Aarhus V, Denmark; 3 University of Rostock, Ulmenstraße 69, 18057 Rostock, Germany; 4 Babson College, Babson Park, FL USA; 5 University of Bath, Bath, UK; 6 Tecnológico de Monterrey, Monterrey, Mexico

**Keywords:** Meta-analysis, Customer engagement marketing, Task-based engagement initiatives, Experiential engagement initiatives, Engagement platforms

## Abstract

As part of their customer engagement (CE) marketing, firms use different platforms to interact with customers, in ways that go beyond purchases. Task-based CE strategies call for customers’ participation in structured, often incentivized tasks; experiential CE initiatives instead aim to stimulate pleasurable experiences for customers. But the optimal uses of these two strategies, in terms of improving customer engagement to produce more positive marketing outcomes, are unclear. With a meta-analysis and data from 395 samples, pertaining to 434,233 customers, the present study develops and tests a unifying framework of how to optimize investments in both two engagement strategies across different engagement platforms. On average, task-based initiatives are more effective in driving customer engagement, but the effects depend on the platform. If platforms support continuous or lean interactions, task-based initiatives are more effective; on platforms that encourage spot interactions, experiential initiatives are preferable. Three customer engagement dimensions (cognitive, emotional, and behavioral) in turn lead to positive marketing outcomes, though in ways that depend on the platforms’ interaction characteristics (intensity, richness, initiation) and differ across digital versus physical platforms. These results provide clear guidance for managers regarding how to plan their CE marketing activities to benefit both their firms and their customers.

**Supplementary information:**

The online version contains supplementary material available at 10.1007/s11747-023-00925-7.

Firms seek to interact with customers in various ways, beyond simple transactions (Pansari and Kumar 2018), and thus exhibit a “deliberate effort to motivate, empower, and measure a customer’s voluntary contribution to its marketing functions, beyond a core, economic transaction” (Harmeling et al., 2017, p. 312). The strategies deployed to stimulate such customer engagement are varied and creative, such as when Lay’s conducts idea contests and asks customers to propose new chip flavors or when Sprite hosted multisensory, live concerts on a New York corner. These strategies also take place on various platforms (e.g., digital vs. physical); Anheuser-Busch spreads its $200 billion annual investment in customer engagement marketing across multiple platforms (Harmeling et al., 2017).

Ultimately though, to be effective, customer engagement (CE) must enhance marketing outcomes such as purchase frequency (ScottGould 2022), an outcome that some observers question (Beckers et al., 2018). Despite the need to clarify which strategies improve customer engagement and when it evokes positive marketing outcomes, previously proposed frameworks pertaining to the antecedents or effectiveness of engagement strategies (e.g., van Doorn et al., 2010; Vivek et al., 2018) tend to be conceptual. Although Harmeling et al. (2017) also identify two primary engagement strategies (task-based and experiential), they do not address different platforms.

More broadly, we find few studies that test for the influences of distinct platform characteristics on the effectiveness of engagement strategies or their marketing outcomes. Whereas Santini et al. (2020) compare the effects on three digital platforms (blogs, Facebook, Twitter), they do not include engagement strategies or platform characteristics,^1^ so one cannot establish whether the effectiveness of engagement strategies depends on the platform and/or which platform characteristics encourage positive marketing outcomes.

Thus, we still lack a unifying framework that provides clear insights into how firms can optimize their investments in engagement strategies. In response, we undertake a meta-analysis of empirical research that links different strategies with CE and integrate insights from both CE marketing and platform theories. In line with an emerging theory of CE marketing (Harmeling et al., 2017), we distinguish two main strategies: *task-based engagement marketing initiatives* that call for customers’ participation in structured, often incentivized tasks to prompt their voluntary contributions (e.g., Lay’s chips idea contests; Harmeling et al., 2017) and *experiential engagement marketing initiatives* that represent attempts to provide pleasurable experiences for customers and thereby motivate their voluntary contributions (e.g., Sprite’s corner concerts; Harmeling et al., 2017). In accordance with platform theory, we also outline the contexts in which CE marketing takes place. Some platforms facilitate interactions with customers better than others (Wichmann et al., 2022); to explain why, we consider factors such as the intensity of interactions that the platforms support (continuous vs. spot), their richness (rich vs. lean), their status (digital vs. physical), and whether the platforms allow the firm or customer to initiate interactions. Table 1 provides examples of typical CE marketing activities across different platforms.

**Table 1:** Examples of CE marketing actions on different engagement platforms

		Continuous Interactions		Spot Interactions	
		Rich Interactions (A)	Lean Interactions (B)	Rich Interactions (C)	Lean Interactions (D)
Digital Platform	Firm-initiated (1)	Sephora’s “Beauty Insider Community” is managed by the firm. Customers can interact with other customers, share make-up experiences (pictures, tips, tricks) and participate in online challenges to experiment with Sephora’s products.	An automated email or in-app push notification from Booking.com asks customers to provide prestructured, text-based feedback regarding each booking. The feedback helps Booking.com improve its services.	The time-limited PlayWithPringles campaign asked customers to produce and upload creative video content (self-recorded, edited, underlaid with music) on TikTok about their Pringles product experience. Customers can interact with other customers and like, share, and comment on the videos.	In a temporary initiative such as Volvo’s Super Bowl #VolvoContest, people have to use the simple hashtag on Twitter and tweet it, whenever they see a car advertisement during the Super Bowl.
	Customer-initiated (2)	An online fan community, such as “IKEA tips, hacks and more,” runs and manages its own Facebook website, publishing different content (videos, images, reels), chat groups, and other forms of interaction around IKEA products. IKEA supports this community and forwards the best customer hacks.	An online fan community such as MacRumors, where members and visitors can submit new text-based rumors about the brand Apple through an email or an anonymous form. Apple supports this community.	A temporary initiative such as the Ice Bucket Challenge, initiated by private activists. Customers created video content, shared it on social media, and interacted with others to generate awareness of ALS disease and encourage donations to the ALS Association.	A one-time initiative, such as a bet by Carter Wilkerson on Twitter, when he asked Wendy’s for a year’s supply of chicken nuggets in exchange for a required number of retweets. His post went viral and broke Ellen DeGeneres’s retweet record. This initiative generated brand awareness for Wendy’s.
Physical Platform	Firm-initiated (3)	Repeated outdoor events for members of the Harley Owners Group managed by Harley Davidson where they can meet, ride together, and share their multisensory riding experiences.	Hilton places a questionnaire in hotel rooms for guests, to encourage them to contribute to the improvement of Hilton’s services and operations.	Temporary indoor events such as Home Depot workshops (e.g., how to build a hanging planter) for customers allow members of the Home Depot community to participate and interact in multisensory workshops.	A temporary initiative such as “text to join” from Rock Creep Tap and Grill (local Canadian restaurant brand) uses simple text flyers on restaurant tables to encourage customers to support a loyalty program.
	Customer-initiated (4)	A fan community organizes the Volkswagen Golf GTI Meeting every year since 1982 in Austria. Participants show their tuned cars, engage in conversations, and participate in different multisensory activities around the brand.	Regular phone calls by an alumni network to inform other alumni about opportunities to financially support the alma mater.	Members of local fan communities (Adidas Runners) self-organize a one-time workshop on meal plans and healthy diets. This event is multisensory and helps members improve their running performance. The communities are supported by Adidas.	A one-time initiative, such as an offline petition of solidarity-minded customers who support the protection of small local business owners (e.g., Annie’s Cambodian Cuisine). Customers are encouraged to sign the petition.

In turn, we can determine that the two strategies mainly affect three CE dimensions, which resonates with the extant conceptualization of CE as a multidimensional construct. Specifically, customer engagement encompasses customers’ volitional investments of their cognitive (e.g., knowledge), emotional (e.g., brand enthusiasm), and behavioral (e.g., skills) resources in interactions with some engagement object (e.g., firm, brand). As Hollebeek et al. (2019, p. 174) explain, because engaged customers tend to “invest more resources in brand interactions than their less engaged counterparts,” they also typically provide deeper and broader contributions to the relationship. As we show, these engaged customers usually invest cognitive resources first, by thinking about and attending to the firm and its brands (Dessart et al. 2016). Then they invest emotional resources, get excited about the firm and its brands, and derive pleasure from the interaction (Hollebeek et al. 2014). Finally, they invest behavioral resources, including referrals and product development ideas.

In addition to the two engagement strategies (task-based and experiential), our meta-analytic framework accounts for two traditional marketing strategies (product performance and brand associations) (Harmeling et al., 2017), which enables us to conduct a comparative assessment of the effectiveness of engagement marketing versus traditional marketing. With our multivariate conceptualization of CE, we also clarify how the strategies influence each of the three CE dimensions, to establish a comprehensive assessment of their effectiveness. With this assessment, we investigate when firms benefit most from engaging customers, such that the CE they evoke translates into marketing outcomes. Finally, we account for platform characteristics, as potential moderators of the relationships of the different strategies with CE dimensions and then with marketing outcomes.

To validate this proposed framework, we employ a meta-analysis of 5,005 correlations reported by 434,233 customers in 395 samples. Meta-analyses can combine and compare results across studies, identify boundaries, and suggest directions for further research (Grewal et al., 2018). Accordingly, we use the vast meta-analytic database we collected to clarify which engagement initiatives are more effective in driving CE and in what conditions. The results suggest that, on average, task-based initiatives are more effective than experiential initiatives, with stronger effects on behavioral and emotional dimensions. Both CE-specific marketing and traditional marketing can stimulate CE, though differences arise with the different CE dimensions. In addition, we specify the increased relevance of task-based initiatives on platforms that support continuous interactions but the dominant influence of experiential initiatives on platforms that support spot interactions. Furthermore, task-based initiatives are more effective on platforms that enable lean rather than rich interactions. In contrast, we find no differences for digital versus physical platforms or for firm- versus customer-initiated interactions, so both strategies appear equally effective in these settings.

Turning to the outcomes of firms’ efforts to engage customers, our analyses suggest that all three CE dimensions relate to behavioral outcomes and intentions, but emotional and cognitive CE exert indirect effects through behavioral CE. Overall, firms benefit from engaging customers, but the positive results depend on the platform used. In detail, behavioral and emotional CE have weaker effects on behavioral intentions on digital (cf. physical) platforms but stronger effects for customer- (cf. firm-) initiated interactions. We find weaker effects of emotional CE on behavioral outcomes for continuous (cf. spot) interactions; the results for rich versus lean interactions vary by the marketing outcome being measured. Finally, cognitive CE exhibits similar effectiveness across all the different platform characteristics. In addition to advancing CE marketing theory and demonstrating the usefulness of integrating platform theory, our findings thus give managers insights into how to leverage different CE strategies and platforms to attain improved marketing outcomes.

## Conceptual background

### Multidimensional conceptualization of CE

Engagement implies a subject who is engaged and an object with which this subject engages, as initially presented in organizational behavior research pertaining to employee engagement (Kahn, 1990) and informed by social identity theory (Ashforth & Mael, 1989). In marketing contexts, the engaged subject refers to existing or prospective customers, and the engagement object might be a brand or firm (Hollebeek & Macky, 2019). Marketing scholars also apply various labels to engagement (e.g., customer brand engagement, digital customer engagement; van Doorn et al., 2010). In turn, different conceptualizations of CE are available (Clark et al., 2020; Web Appendix B). In general, this variety can be summarized in two schools of thought: a *behavioral conceptualization* that focuses on behavioral CE exclusively (Kumar et al., 2019) or a *multidimensional conceptualization* that spans behavioral and psychological CE components (Clark et al., 2020). But even within these schools of thought, authors emphasize different substantive content (e.g., which dimensions comprise CE) and structures (e.g., relationships among CE dimensions).

That is, behavioral conceptualizations tend to suggest that recommendation, influence, and feedback behaviors drive firm-related outcomes and performance (Pansari & Kumar, 2017; van Doorn et al., 2010). However, scholars who adopt a value-based perspective broaden this scope to include transactional (e.g., customer purchases) in addition to nontransactional (e.g., word-of-mouth) contributions (Beckers et al., 2018). In line with the narrow conceptualization, we argue that only behaviors that go beyond transactions represent CE (van Doorn et al., 2010), so the goal should be to stimulate nontransactional, behavioral CE. Following this reasoning, in considering behavioral CE, we focus on nontransactional behaviors; transactional behaviors represent outcomes.

The multidimensional view of CE instead has been informed by organizational research (Rich et al., 2010), in which employees’ work engagement comprises cognitive, emotional, and behavioral CE (Hollebeek et al., 2019). As we noted previously, in consumer settings, CE refers to investments of resources, such as cognitive (e.g., knowledge), emotional (e.g., enthusiasm), and behavioral (e.g., skills) resources in specific interactions with a firm or brand. Engaged customers invest cognitive resources first, followed by emotional and behavioral investments (Hollebeek et al., 2014; Oliver, 1999). Attitudinal theories also provide relevant predictions of how cognitive and emotional constructs can influence behavior (Fishbein & Ajzen, 1975). Thus, we anticipate that cognitive CE relates to emotional CE, which influence behavioral CE.

### CE marketing

Prior studies have traced the evolution of marketing, from transactional to relationship to CE marketing (Harmeling et al., 2017; Verhoef et al., 2010). That is, initial firm relationships with customers prioritized transactions (e.g., purchases), but firms’ focus on such transactional value creation shifted, prompting efforts that seek to establish and maintain customer relationships (Palmatier & Steinhoff, 2019; Pansari & Kumar, 2018). Customers appreciate connections with firms, which can occur through various platforms, as well as interacting with other customers (Pansari and Kumar 2018). Beckers et al. (2018) predict deeper relationships with engaged customers, who interact with the former at various points, beyond the point of purchase. During such interactions, customers form opinions about and become attached to the firm and its brands (Fuchs et al., 2010). Some engagement studies refer to the service-dominant (S-D) logic (Vargo & Lusch, 2004); this stream of literature emphasizes value co-creation and customers’ nontransactional contributions (Brodie et al., 2011; Payne et al., 2008). Even as the interrelationship of CE and the S-D logic has been established (Brodie et al., 2011), only recently have scholars begun developing integrated, conceptual frameworks (Hollebeek et al., 2019).

Regarding the antecedents of CE, van Doorn et al. (2010) propose three categories: customer (e.g., identity, consumption goals), firm (e.g., brand characteristics, firm reputation), and context (e.g., competitive factors). Harmeling et al. (2017) focus on firm strategies and consolidate insights from different literature streams to define and classify CE-specific marketing strategies, based on whether the strategies (1) deliberately motivate customers to invest resources and engage with the firm (i.e., CE marketing) or (2) organically evoke CE, without deliberate effort by the firm (i.e., traditional marketing strategies). In addition, they clarify the two types of engagement strategies that we cited previously. Due to their promising outcomes, task-based initiatives were the focus of many early studies. They assign structured tasks to customers (e.g., write a review, refer a friend), which customers must invest mental and physical effort to complete. In Fuchs and Schreier’s (2011) study of how task-based initiatives influence participation in new product development, they identify customers’ sense of psychological ownership, stemming from the effort they invest, as a source of more favorable evaluations of the firm (see also Van Dyne & Pierce, 2004). In contrast, experiential initiatives often feature playful events, rather than work-like tasks, that can induce a sense of self-transformation and encourage customers to incorporate the brand into their self-concept (Markus & Kunda, 1986). These initiatives strengthen psychological and emotional connections. For example, Cova and Pace (2006) show how Ferrero’s experiential events, such as Nutella Parties, prompted customers’ identification with the “my Nutella Community.” However, relatively fewer studies have examined this type of strategy.

We also acknowledge that traditional marketing strategies might organically stimulate Harmeling et al. (2017) stress the importance of *product performance* and *brand associations*, with the prediction that when customers have excellent experiences with an offering’s performance or the brand, they want to support the firm (e.g., through word-of-mouth), beyond purchasing (Verleye et al., 2014; Wallace et al., 2014).

Thus, in our proposed framework, we include task-based and experiential engagement strategies, together with traditional marketing strategies, so that we can compare their effectiveness. In some ways, our framework follows Harmeling et al.’s (2017), but we note several key differences. Their framework features behavioral CE only, it ignores platform differences, and excludes marketing outcomes. Moreover, we analyze the influence of a larger set of antecedents (Web Appendix C). In line with suggestions that CE can influence marketing outcomes, including firm performance (Kumar & Pansari, 2016) and customers’ behavioral intentions (Santini et al., 2020), we include these considerations in our framework.

### Platform theory

Platforms enable and facilitate interactions between two or more parties, such as customers and firms (Rangaswamy et al., 2020; Wichmann et al., 2022). They function to sell goods and services, support socializing, and exchange information (Bonina et al., 2021). Platform theory distinguishes physical and digital platforms (Breidbach & Brodie, 2016; Wirtz et al. 2019). The Consumer Electronic Show is a physical platform that allows exhibitors and potential customers to interact directly, whereas Amazon or eBay provide digital platforms that facilitate online seller–buyer interactions (Breidbach & Brodie, 2016). Wirtz et al. (2019) also differentiate types of digital platforms, such as search (e.g., Bing), communication (e.g., WhatsApp), social media (e.g., Facebook), and matching (e.g., TaskRabbit) platforms. In their literature review, Rangaswamy et al. (2020) stress that despite the many studies of platforms in general, few of them elucidate the role of marketing (e.g., Bhargava & Rubel, 2019; Lamberton & Rose, 2012; Rosario et al., 2016). Thus, we need more research that takes a marketing perspective on platforms and tests their characteristics.

In customer engagement literature, studies often refer to “engagement platforms” that enable customer–firm or customer–customer interactions (Blasco-Arcas et al., 2016; Breidbach & Brodie, 2016). Digital engagement platforms tend to include review sections, brand development co-creation spaces, and social network elements (Blasco-Arcas et al., 2016). They allow customers to engage with a focal object (e.g., brand) during the purchase process, so firms in turn can actively drive customer engagement through their CE marketing efforts, such as encouraging customers to create content and exchange with others (Wichmann et al., 2022). Yet we find few studies examining the influence of platform characteristics on the effectiveness of engagement strategies, possibly because most research in this domain features a single sample on one platform and thus cannot undertake a comparative assessment. Santini et al. (2020) compare CE effects across blogs, Facebook, and Twitter, but they do not address engagement strategies or platform characteristics.

Finally, platform theory also suggests that some platforms are better suited to facilitate interactions than others. Specifically, prior literature identifies four main platform characteristics, which we accordingly include as moderators in our framework. That is, firms might leverage platforms with varying interaction intensity (continuous vs. spot) and richness (rich vs. lean) (Kaplan & Haenlein, 2010; Sawhney et al., 2005). They might also use digital or physical platforms to access customers, and they can consider whether the firm or customers initiate interactions the platforms they use (Beckers et al., 2018; Meire et al., 2019).

## Meta-analytic framework

**Fig. 1:**
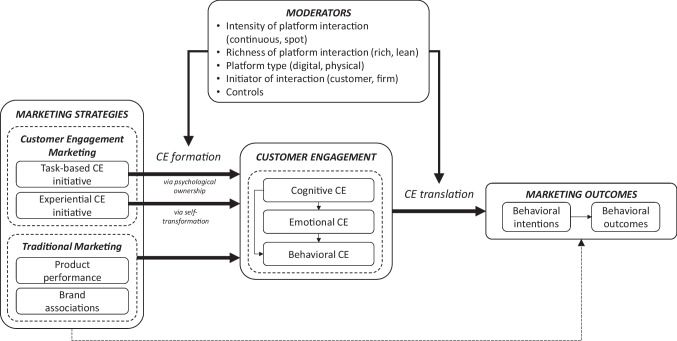
Meta-analytic framework

Like Blut et al. (2021), we choose not to derive formal, main effects hypotheses; instead, we present a summary and outline how our findings help resolve discrepancies in prior research (e.g., differential effects of strategies on CE). We briefly explain the underlying mechanisms of different CE strategies. Because of their novelty though, we derive hypotheses for the moderating effects of platforms characteristics (see Fig. 1).

### Antecedents: CE marketing initiatives

The influences of task-based versus experiential initiatives on CE likely move through two distinct mechanisms: psychological ownership and self-transformation.^2^ First, because task-based initiatives require customers to invest some mental effort to perform a specific task (e.g., idea competition), they may induce a sense of psychological ownership (Harmeling et al., 2017), defined as “possessive feelings that some [engagement] object is ‘MINE’” (Van Dyne & Pierce, 2004, p. 440). This sense of ownership also evokes a feeling of responsibility toward the engagement object (Morewedge et al., 2021). Therefore, customers feel motivated to live up to their responsibility by supporting the firm, that is, by investing their cognitive, emotional, and behavioral resources in interactions with it.

Second, experiential initiatives, which tend to be multisensory, highly participatory, and shared (Harmeling et al., 2017), can lead to self-transformation and shifted customer beliefs and attitudes (Schouten et al., 2007), such that customers incorporate the brand that has provided the experience into their self-concept (Markus & Kunda, 1986). Once a firm or brand is incorporated into customers’ self-perceptions, they likely support it by investing cognitive, emotional, and behavioral resources in interactions (Harmeling et al., 2017).

Prior research indicates that it is easier to induce psychological ownership feelings through task-based initiatives than self-transformation through experiential initiatives (Harmeling et al., 2017). Self-transformation processes are complex and difficult to trigger because a customer’s self is relatively stable and resistant to change (Markus & Kunda, 1986). Accordingly, we anticipate that task-based initiatives have stronger effects on all three CE dimensions than experiential initiatives do.

### Antecedents: Traditional marketing

Product performance (i.e., product-related experiences with the core offering) and brand associations (i.e., brand-related experiences with the core offering) can stimulate CE. Perceptions of the firm’s core offering, developed over time, often get stored in customers’ minds (Anderson & Bower, 2014; Harmeling et al., 2017; Keller, 1993), and when these positive experiences are recalled, customers are more likely to engage with the firm. Such product and brand knowledge may trigger cognitions, emotions, and behaviors (Anderson & Bower, 2014), signaling their positive links to the three dimensions of CE (Dessart et al., 2016). That is, due to their past positive experiences, customers should be motivated to invest cognitive, emotional, and behavioral resources into interactions with the firm or brand.

Finally, we predict differential effectiveness of CE versus traditional marketing. If customers can identify an underlying motive for firms’ strategies, they find the marketing efforts less credible and convincing (Roehm & Brady, 2007). The CE initiatives deliberately exist to push customers’ resource contributions, but traditional marketing strategies prioritize positive product and brand experiences, with engagement as an organic outcome (Harmeling et al., 2017). Thus, traditional strategies should be more credible and effective for driving CE.

### Consequences of CE

The separate CE dimensions likely evoke different marketing outcomes, which might include behavioral intentions toward a firm (e.g., intention to repurchase; van Doorn et al., 2010) or behavioral outcomes (e.g., actual repurchases; Beckers et al., 2018). Engaged customers invest their cognitive, emotional, and behavioral resources into specific interactions and perceive their efforts to support the firm as rewarding, such that they should be eager to remain in a relationship with the firm. We also expect dependencies between intentions and behavior, as suggested by attitudinal theories (Fishbein & Ajzen, 1975) and prior engagement literature (Hollebeek et al., 2014).

### Platform characteristics as moderators

Platform characteristics arguably might inform both the formation of CE and its translation into marketing outcomes. We propose that all platform characteristics likely moderate the translation of CE into marketing outcomes, but only two of them matter for the formation of CE. Furthermore, though the effectiveness of task-based initiatives depends on customers’ understanding of the assigned task, experiential initiatives are more effective when customers experience unusual, emotionally intense, interactive events (Harmeling et al., 2017). As we discuss next, prior literature implies that the intensity and richness of platform interactions moderate the effects of CE initiatives (Kaplan & Haenlein, 2010).

#### Intensity of platform interaction

Continuous interactions differ from spot interactions in their frequency and repetitiveness (Sawhney et al., 2005). Regarding this platform characteristic, we posit that task-based initiatives exert stronger effects on CE dimensions in continuous than in spot interactions, whereas experiential initiatives may have weaker effects. For example, customers can interact continuously with Polaroid on its brand-owned platform, such as by uploading pictures, sharing content, and referring friends (www.polaroid.com). Through these ongoing interactions, customers gain a better understanding of any assigned tasks and learn more, which—according to CE marketing theory (Harmeling et al., 2017)—should induce stronger feelings of ownership. But when Absolut Vodka introduced “out-of-this-world” experiential initiatives, it sought to evoke immediate emotional and psychological reactions to each event. These transformative experiences likely increased customers’ sense of emotional intensity, which fuel self-evaluative cognitive processes (Harmeling et al., 2015). If they were to repeat such spectacular interactions frequently though, it would become more difficult to induce emotions due to wear-out effects (Sharot et al., 2004). It is difficult to surprise and excite customers on an ongoing basis (Maguire et al., 2011). Thus, experiential initiatives likely require spot interactions to drive CE, whereas task-based initiatives benefit from continuous interactions.

#### H1

The positive relationships of task-based initiatives with CE dimensions are stronger for continuous than for spot interactions.

#### H2

The positive relationships of experiential initiatives with CE dimensions are weaker for continuous than for spot interactions.

In turn, we predict stronger relationships between CE dimensions and marketing outcomes in spot compared with continuous interactions. Santini et al. (2020) explain that the strength of CE effects on marketing outcomes depends on the personal relevance of the engagement object to customers. When personal relevance is greater, customers likely consider their investments in interacting with the engagement object when they reflect on their future behavioral intentions or behaviors (Santini et al., 2020). The engagement object is more salient in spot than in continuous interactions. That is, as Johnston and Lane (2021, p. 2) explain, spot interactions occur “over short periods of time when an organization seeks to affect or connect with its stakeholders around a focal topic (e.g., issue or decision).” Because these spot interactions limited in time and specific to some particular issue or topic, they are more instrumental too (Johnston & Lane, 2018). For example, the Ice Bucket Challenge created vast awareness of the need for more research into ALS through intensive communication on social media within just three months. Customers likely reflect on the importance of this engagement object (Santini et al., 2020), so the effects of CE dimensions on marketing outcomes should be stronger for spot than for continuous interactions.

#### H3

The positive relationships of CE dimensions with (a) behavioral intentions and (b) behavioral outcomes are weaker for continuous than for spot interactions.

#### Richness of interaction

The platform interaction richness aligns with the broader concept of media richness, which establishes that “media differ in the degree of richness they possess—that is, the amount of information they allow to be transmitted in a given time interval” (Kaplan & Haenlein, 2010, p. 61). Rich platform interactions differ from lean ones in the number and diversity of informational cues they transmit (e.g., videos vs. images vs. text; Cao et al., 2021; Dennis & Kinney, 1998). Whereas wikis and Twitter are relatively lean engagement platforms, social games and social worlds (e.g., Facebook’s metaverse) represent rich engagement platforms (Kaplan & Haenlein, 2010). Media richness theory acknowledges the importance of considering the capacity of different communication media to process “rich” information but does not offer predictions about the effects of these differences (Dennis & Kinney, 1998). Thus, we propose two rival hypotheses.

First, rich media might enhance communication effectiveness, because the greater amount and diversity of information transmitted improve understanding of the message (Vickery et al., 2004). Therefore, rich media should generally outperform lean media in terms of communication effectiveness. With respect to CE initiatives, both task-based and experiential initiatives could have stronger effects in rich interactions, in which companies communicate diverse information cues that help customers understand task-based initiatives, and if customers understand their assigned task, they likely develop feelings of ownership (Harmeling et al., 2017). Moreover, rich interactions may help customers incorporate the firm into their self-perceptions in response to experiential initiatives (Kaplan & Haenlein, 2010). Experiential events gain transformational capacities when they are unusual, emotionally intense, and interactive (Harmeling et al., 2017). Rich interactions offer more opportunities to communicate what makes an event unusual, emotionally intense, and interactive.

Second, rich media are not always better than lean media (Dennis & Kinney, 1998). According to the task–media fit hypothesis, unstructured tasks require rich media that can carry different types of messages, but structured tasks require lean media (Shirani et al., 1999). Task performance improves when there is a “match between information requirements of the task and a medium’s ability to convey information richness” (Suh, 1999, p. 297). If a medium is too rich for the task, it may distract customers and lead to inefficiency, because some of the rich information provided is not essential for effective communication (Suh, 1999). With respect to CE, task-based initiatives involve requests for structured tasks (e.g., retweet a hashtag) that guide customers’ voluntary resource contributions (Harmeling et al., 2017). To send such specific instructions, leaner media may be better suited. McGrath and Hollingshead (1993) stress, in their task–media fit framework, that leaner media (e.g., text-based) facilitate structured tasks, such as generating new product ideas and plans, better than richer media (e.g., virtual world). If customers get distracted by rich information that hinders their understanding of the assigned task, they are less likely to develop feelings of ownership. Thus, we acknowledge the argument that task-based initiatives may be less effective in rich interactions. We do not propose a rival hypothesis for experiential initiatives. According to Harmeling et al. (2017), experiential initiatives resemble unstructured play, and thus richer media still would be better suited according to the task–media fit hypothesis.

#### H4

The positive relationships of (a) task-based initiatives and (b) experiential-based initiatives with CE dimensions are stronger for rich than for lean interactions.

#### H5

The positive relationships of task-based initiatives with CE dimensions are weaker for rich than for lean interactions.

Finally, the richness of the platform interaction should influence the translation of CE into marketing outcomes, because rich interactions might disrupt the effects of CE dimensions on marketing outcomes. The diverse informational cues they transmit tend to increase the complexity of customers’ decision-making (Isen, 2001; Malhotra, 1984). In such a situation, customer’s own sense of engagement, becomes less important.

#### H6

The positive relationships of CE dimensions with (a) behavioral intentions and (b) behavioral outcomes are weaker for rich than for lean interactions.

#### Digital versus physical platform

Platform theory suggests that digital platforms (e.g., social networking sites) differ from physical platforms (e.g., outdoor events). Lieberman and Schroeder (2020) explain that, relative to offline interactions, online interactions provide more opportunities to form new social ties, disseminate information widely, and achieve anonymity. However, the effects of digital versus physical platforms on CE are uncertain, so we propose two rival hypotheses.

On the one hand, CE dimensions should display stronger effects on marketing outcomes on digital platforms, where CE is more relevant, due to the structural differences between digital and physical platforms (Lieberman & Schroeder, 2020). On digital platforms, customers can access information about the engagement object and enter into exchanges with others easily (Lieberman & Schroeder, 2020; Santini et al., 2020). For example, CE gained great popularity with the rise of social media, such as Twitter, Instagram, and Facebook, which facilitate the formation and maintenance of social networks (Lieberman & Schroeder, 2020). Through facilitated exchanges and information dissemination on digital platforms, customers learn about the importance of the engagement object (Santini et al., 2020), so CE should drive customers’ future marketing outcomes on digital platforms.

On the other hand, customers often rely on stereotypes when assessing computer-mediated communication (Jacobson, 1999) and perceive exchanges on digital platforms as shallow or lacking in depth (Taylor, 2022), as well as less meaningful than real-world exchanges. For example, after the peak of the COVID-19 pandemic, many firms encouraged employees to return to offices, citing the greater quality of real-world interactions compared with virtual interactions. Furthermore, Taylor and Kent (2014, p. 393) stress that “[p]osting comments on a social media site is no substitute for calling someone on the telephone, or meeting others, to discuss an issue.” Accordingly, exchanges about a focal issue (or CE effects) on a physical platform may be perceived as more meaningful than exchanges about the same issue on a digital platform.

#### H7

The positive relationships of CE dimensions with (a) behavioral intentions and (b) behavioral outcomes are stronger for digital than for physical platforms.

#### H8

The positive relationships of CE dimensions with (a) behavioral intentions and (b) behavioral outcomes are weaker for digital than for physical platforms.

#### Initiator of interaction

As proposed by platform theory, we also consider the effects of the initiator of platform interactions, which may moderate the translation of CE dimension into marketing outcomes. Vivek et al. (2012, p. 132) explain that “either the provider (or organization or firm) or the customer may initiate the interaction”; we propose stronger effects of CE dimensions on marketing outcomes for customer-initiated interactions, due to their reflection of the importance of the focal issue to customers (Beckers et al., 2018). Usually, customers initiate an interaction when experiencing an urge to exchange about an issue with great relevance for them (Paluch & Blut, 2013). Comparing customer- with firm-initiated brand communities, Li et al. (2019) argue that the instigator influences the importance of the engagement object. When customers make decisions, they tend to consider personally relevant factors, which in turn inform whether they decide to remain in a relationship with the firm. Thus, the CE dimensions should display stronger relationships with marketing outcomes when the interactions are initiated by the customer than the firm.

#### H9

The positive relationships of CE dimensions with (a) behavioral intentions and (b) behavioral outcomes are stronger for customer- than firm-initiated interactions.

### Traditional marketing strategies

The effects of product performance and brand associations, as traditional marketing efforts, also might vary with platform characteristics. Our focus explicitly is on CE initiatives, so we do not derive hypotheses for these traditional marketing strategies. Instead, we briefly explain a rationale for these parallel moderating effects. For both product performance and brand associations, we anticipate stronger effects of continuous versus spot interactions. Citing knowledge structures, Harmeling et al. (2017) note that repetition strengthens cognitive bonds, which improves information recall (Anderson & Bower, 2014). Repetition, as facilitated by continuous interactions, helps customers activate and consolidate memories (Pezzulo et al., 2014), such that they are more likely to recall past positive experiences, which can drive CE. In addition, product performance and brand associations both might exert weaker effects on CE dimensions in rich interactions, because their diverse informational cues distract customers (Dennis & Kinney, 1998), who then are less likely to pay attention to any specific cue that evokes their past positive experience with the firm. Because customers are less likely to recall past positive experiences in rich interactions, compared with lean ones, they are less likely to engage. We do not expect any differences for digital versus physical platforms or for different initiators of the interaction.

### Control variables

We include some methodological considerations in our framework, due to our need to account for the diversity of the studies we collected in our meta-analysis. First, we check for differences between customer surveys and other research designs. Surveys usually produce smaller effect sizes than experiments, because it is more difficult to eliminate potential confounds (Grewal et al., 2018). Second, the data collection approaches adopted by our source articles might involve single or multiple industries. With more diverse industries, the range of constructs of interest increases, which influences the magnitude of the effect sizes (Geyskens et al., 1998). Third, we consider the quality of the publication outlet. High-quality journals have more rigid mechanisms to control for factors that might inflate effect sizes (e.g., common method variance) (Hunter & Schmidt, 2004). Fourth, publication status serves as a moderator; we distinguish between published and unpublished contributions. Insignificant effects are less likely to be published in journals, due to publication bias (Hunter & Schmidt, 2004). Fifth, we include the study year, because customers’ greater experience over time with social media and other technology may foster their engagement (Hollebeek et al., 2014).

## Method

### Search strategy and inclusion criteria

We searched for CE studies in several electronic databases (ABI/INFORM, ProQuest, Web of Science, EBSCO Information Services), using combinations of keywords such as “customer engagement,” “consumer engagement,” and “customer brand engagement.” We complemented these efforts with additional searches through Google Scholar, to identify conference proceedings and dissertations not published in journals. We also identified studies that cited van Doorn et al. (2010), as an influential contribution in this research domain, and reviewed the reference lists of all those studies, as well as those of other review articles, such as Santini et al. (2020) review. Finally, we searched for and included unpublished data sets.

Four criteria determine whether studies enter the meta-analysis. First, we required that they be empirical studies at the customer level and thus excluded conceptual articles (Hollebeek et al., 2019), qualitative studies (Hollebeek, 2013), and empirical studies that investigate engagement at the firm level (Gambetti et al., 2012). Second, the articles had to measure the CE construct; if they refer to CE but do not measure it, we exclude them. Third, eligible articles contain sufficient data to calculate effect size statistics (e.g., correlation coefficients, regression weights, t-values) for at least one relationship between CE and another construct. Fourth, we excluded studies of political engagement, civic engagement, or digital literacy, to ensure the study contexts involve firms and their products. We obtained 350 usable studies for the meta-analysis.

### Effect size measures and data coding

We used correlation coefficients as effect sizes because they are independent of the scale and reported in most studies. If this information was not available, we converted other statistical information into correlations (Peterson & Brown, 2005). In line with conventional standards for meta-analyses, we averaged the effect sizes of samples that reported more than one correlation for the same relationship, to avoid giving any sample too much weight in subsequent analyses (Palmatier et al., 2006). After averaging the effect sizes, the data set included 5,005 correlations, reported in 395 independent samples extracted from 350 studies. Of the 395 samples, 325 were published in journals and 70 in conference proceedings, dissertations, or unpublished works; 10 were published between 2005 and 2010, 101 between 2011 and 2015, and 284 between 2016 and 2020. The cumulative sample size was 434,233. In more detail, coders initially extracted 7,323 effect sizes, which were averaged into 5,005, of which 1,612 effect sizes involve constructs in Fig. 1, and the rest pertain to the antecedents discussed in the Web Appendix C.

Three coders extracted necessary information and calculated the effect sizes. They also classified the effects according to the construct definitions in Table 2 and examined the scale items to classify the effect sizes. They reached 97% agreement and discussed any disagreements jointly. The coders extracted information about sample sizes, construct reliabilities, study characteristics that reflect the contextual setting of the study, and method characteristics. With dummy codes, they gauge the four focal moderators: interaction intensity (1 = continuous; 0 = spot), richness (1 = rich; 0 = lean), platform type (1 = digital; 0 = physical), and initiator (1 = customer; 0 = firm). They also dummy-coded the control variables: data collection (1 = single industry; 0 = multiple), research design (1 = survey; 0 = other), and publication status (1 = published; 0 = unpublished). We extracted the study year from the articles and assessed publication outlet quality according to the ABS journal list, with ratings ranging from 1 (low quality) to 4 (high quality).

**Table 2:** Construct definitions, aliases, and representative studies

Construct	Definition	Common aliases	Representative studies
Antecedents			
Task-based initiatives	Firm initiatives to motivate customers to complete a structured task and make voluntary contributions to marketing functions, beyond purchasing a product (Harmeling et al., 2017).	Incentives, referral campaign, referral program, rewards, word-of-mouth program	Harmeling et al. (2017); Beckers et al. (2018); Meire et al. (2019)
Experiential initiatives	Firm initiatives focusing on the generation of positive emotions to intrinsically motivate customers to contribute to marketing functions, beyond purchasing a product (Harmeling et al., 2017).	Emotional marketer-generated content, event marketing, experiential marketing	Harmeling et al. (2017); Meire et al. (2019)
Product performance	Customers’ product-related experiences with the firm’s core offering, such as quality assessments (Harmeling et al., 2017).	Perceived quality, product quality, service quality, system quality, website service quality	Ho et al. (2020); Verleye et al. (2014)
Brand associations	Customers’ brand-related experiences with the firm’s core offering, such as brand reputation (Harmeling et al., 2017).	Brand awareness, brand equity, brand image, brand reputation	Groeger et al. (2016); Wirtz (2013)
CE dimensions			
Cognitive CE	Extent to which customers invest cognitive resources into the interaction with an engagement object (Hollebeek, 2011). It is characterized by customers spending time thinking about the engagement object and paying attention to it (Hollebeek et al. 2014).	Absorption, attention, cognitive engagement, cognitive processing	Hepola et al. (2017); Hollebeek et al. (2014); Leckie et al. (2016)
Emotional CE	Extent to which customers invest emotional resources into the interaction with an engagement object (Hollebeek, 2011); it is characterized by consumers’ enthusiasm about the engagement object.	Affection, dedication, emotional engagement, enthusiasm	Hepola et al. (2017); Hollebeek et al. (2014); Leckie et al. (2016)
Behavioral CE	Extent to which customers invest behavioral resources into the interaction with the engagement object (Hollebeek, 2011; Pansari & Kumar, 2017). Customers display different voluntary behaviors, such as content contributions, ratings, sharing, commenting, and helping others (Hollebeek et al. 2014).	Activation, behavioral engagement, commenting, content consumption, content contribution, engagement activity, participation, reading, recommending, sharing, vigor	Groeger et al. (2016); Hepola et al. (2017); Hollebeek et al. (2014); Leckie et al. (2016)
Consequences			
Behavioral intentions	Positive intentions toward a firm, such as intentions to repurchase (Dick & Basu, 1994).	Continuance intention, purchase intention, loyalty intentions, repurchase intention, usage intention	Algesheimer et al. (2005); Fang (2017)
Behavioral outcomes	Actual behaviors and actions toward a firm, such as purchase or repurchase behavior and preferences for the firm’s products or services over others (Dick & Basu, 1994).	Behavioral loyalty, purchase, repurchase behavior, sales, customer spending, product usage	Dessart (2017); Groeger et al. (2016)

### Integrating effect sizes and multivariate analyses

We used the widely adopted random effect approach suggested by Hunter and Schmidt (2004) to integrate effect sizes. First, we corrected the effect sizes for artifacts, including measurement error in the dependent or independent variable, then divided the correlations by the square root of the product of the respective reliabilities of the two constructs of interest. Second, we weighted the measurement error-corrected correlations by the sample size to correct for sampling errors. Third, with 95% confidence intervals for each sample size–weighted and artifact-adjusted correlation, we checked the power of our statistical tests (Muncer et al., 2003). We also calculated credibility intervals, which indicate the distribution of effect sizes (Hunter & Schmidt, 2004). Wide credibility intervals suggest variation in effect sizes and the need for moderator analyses to account for unexplained variance. Fourth, to assess the homogeneity of the effect size distribution, we used the χ^2^ test of homogeneity (Hunter & Schmidt, 2004). Fifth, as a check for potential publication bias, we used Rosenthal’s (1979) fail-safe N (FSN), which indicates the number of studies with null results that would be required to lower a significant relationship to a barely significant level (*p* = 0.05). Rosenthal (1979) suggests a tolerance level, such that the results are robust when FSNs are greater than 5 ⋅ *k* + 10, where *k* equals the number of correlations. As another check for publication bias, we used funnel plots with effect sizes on one axis and sample sizes on the other; an asymmetric plot would indicate potential publication bias. Sixth, we calculated the shared variances between constructs and the binomial effect size display (BESD), to determine the practical relevance of the meta-analytic findings. A high BESD between CE and behavioral intentions indicates the likelihood of more favorable outcomes from one group (e.g., engaged customers) relative to a reference group (e.g., disengaged customers) (Grewal et al., 2018). Following these checks, we analyzed the data using a multilevel moderator analysis and structural equation modeling. Web Appendix D contains more information about the multivariate analyses.

## Results

### Descriptive results

Table 3 contains the results of the effect size integration. In the meta-analytic framework in Fig. 1, CE is a multidimensional construct, but to acknowledge alternative CE conceptualizations (Web Appendix E), we report the results for a unidimensional and higher-order conceptualization too. The effect size integration results consistently suggest the use of our proposed CE conceptualization, due to the observable differences among CE dimensions. Thus, we only discuss the results of the multidimensional approach. The shared variances among CE dimensions are rather low (29–48%), which points to three separate, lower-order constructs rather than one higher-order or unidimensional conceptualization (Edwards, 2001). We test for differences using multilevel modeling, and the results support the proposed conceptualization (Web Appendix F).

**Table 3:** Descriptive results for CE antecedents and consequences

Relationship		k	N	rwc	R^2^	BESD	CI_low_	CI_high_	CR_low_	CR_high_	Q	FSN	Power
Antecedents													
CE marketing													
Task-based initiatives → Cognitive CE		15	2863	0.37*	14%	2.17	0.26	0.48	0.11	0.63	98*	1132	> 0.999
Task-based initiatives → Emotional CE		18	5637	0.48*	23%	2.85	0.39	0.56	0.26	0.70	130*	4020	> 0.999
Task-based initiatives → Behavioral CE		48	35,495	0.21*	4%	1.53	0.14	0.28	− 0.10	0.52	1596*	26,535	> 0.999
Task-based initiatives → Unidimensional CE		11	4242	0.54*	29%	3.35	0.43	0.65	0.32	0.76	96*	3412	> 0.999
Task-based initiatives → Higher-order CE		4	942	0.29*	8%	1.82	0.08	0.49	0.04	0.53	28*	63	> 0.999
Experiential initiatives → Cognitive CE		11	1442	0.38*	14%	2.23	0.32	0.44	0.38	0.38	9	430	> 0.999
Experiential initiatives → Emotional CE		11	1442	0.38*	14%	2.23	0.33	0.44	0.38	0.38	8	450	> 0.999
Experiential initiatives → Behavioral CE		33	26,824	0.12*	1%	1.27	0.03	0.20	− 0.19	0.42	1109*	8936	> 0.999
Experiential initiatives → Unidimensional CE		5	1521	0.50*	25%	3.00	0.29	0.70	0.21	0.79	59*	530	> 0.999
Experiential initiatives → Higher-order CE		11	1439	0.38*	14%	2.23	0.22	0.54	0.05	0.71	76*	428	> 0.999
Traditional marketing													
Product performance → Cognitive CE		33	8754	0.50*	25%	3.00	0.44	0.56	0.29	0.70	184*	16,809	> 0.999
Product performance → Emotional CE		25	7245	0.60*	36%	4.00	0.55	0.65	0.44	0.76	96*	15,302	> 0.999
Product performance → Behavioral CE		60	21,340	0.47*	22%	2.77	0.42	0.51	0.23	0.70	589*	67,232	> 0.999
Product performance → Unidimensional CE		15	6115	0.50*	25%	3.00	0.38	0.62	0.20	0.79	247*	5746	> 0.999
Product performance → Higher-order CE		11	2120	0.23*	5%	1.60	0.14	0.33	0.05	0.42	42*	261	> 0.999
Brand associations → Cognitive CE		31	9393	0.58*	34%	3.76	0.51	0.66	0.32	0.84	292*	21,225	> 0.999
Brand associations → Emotional CE		23	6023	0.59*	35%	3.88	0.53	0.65	0.41	0.77	98*	10,880	> 0.999
Brand associations → Behavioral CE		63	26,219	0.41*	17%	2.39	0.35	0.46	0.12	0.69	1001*	70,817	> 0.999
Brand associations → Unidimensional CE		19	6847	0.53*	28%	3.26	0.40	0.67	0.17	0.90	427*	8220	> 0.999
Brand associations → Higher-order CE		17	3734	0.65*	42%	4.71	0.56	0.74	0.43	0.87	87*	6437	> 0.999
Consequences													
Cognitive CE → Behavioral intentions		57	19,767	0.50*	25%	3.00	0.46	0.55	0.28	0.73	492*	71,950	> 0.999
Emotional CE → Behavioral intentions		54	18,175	0.55*	30%	3.44	0.49	0.61	0.27	0.83	684*	75,433	> 0.999
Behavioral CE → Behavioral intentions		113	48,648	0.49*	24%	2.92	0.44	0.53	0.19	0.79	2074*	323,108	> 0.999
Unidimensional CE → Behavioral intentions		33	14,501	0.58*	34%	3.76	0.50	0.65	0.29	0.86	530*	37,271	> 0.999
Higher-order CE → Behavioral intentions		21	4654	0.67*	45%	5.06	0.61	0.73	0.50	0.84	71*	11,386	> 0.999
Cognitive CE → Behavioral outcome		16	3802	0.21*	4%	1.53	0.14	0.27	0.07	0.35	47*	743	> 0.999
Emotional CE → Behavioral outcome		14	3634	0.28*	8%	1.78	0.21	0.35	0.12	0.44	53*	1113	> 0.999
Behavioral CE → Behavioral outcome		44	28,149	0.24*	6%	1.63	0.17	0.30	− 0.05	0.52	1037*	21,668	> 0.999
Unidimensional CE → Behavioral outcome		8	2576	0.32*	10%	1.94	0.21	0.43	0.14	0.50	46*	570	> 0.999
Higher-order CE → Behavioral outcome		13	6612	0.27*	7%	1.74	0.13	0.40	− 0.05	0.58	299*	2901	> 0.999

k = number of effect sizes, N = cumulative sample size, rwc = sample-weighted, reliability-adjusted average correlation, R^2^ = shared variance, BESD = binomial effect size display, CI = 95% confidence interval, CR = 80% credibility interval, Q = Q statistic, FSN = fail-safe N, Power = results of power test. * *p* < 0.05

#### CE marketing initiatives

As we detail in Table 3, both task-based and experiential initiatives exert significant effects on all CE dimensions, though these effects differ by dimension. The task-based initiatives have weak effects on behavioral CE (sample-weighted, reliability-adjusted average correlation [rwc] = 0.21, *p* < 0.05) but stronger effects for cognitive CE (rwc = 0.37, *p* < 0.05; Z_BCE−CCE_ = 9.00, *p* < 0.01) and emotional CE (rwc = 0.48, *p* < 0.05; Z_BCE−ECE_ = 21.31, *p* < 0.01). We find larger effect sizes of experiential initiatives for cognitive CE (rwc = 0.38, *p* < 0.05; Z_BCE−CCE_ = 10.42, *p* < 0.01) and emotional CE (rwc = 0.38, *p* < 0.05; Z_BCE−ECE_ = 10.67, *p* < 0.01) than for behavioral CE (rwc = 0.12, *p* < 0.05). These latter findings may suggest indirect effects of both initiatives, through cognitive and emotional CE. Comparing both engagement initiatives, we find further differences. Task-based initiatives, on average, are more effective in driving CE than experiential initiatives, according to their stronger effect sizes on behavioral CE (rwc_task_ = 0.21, rwc_exp__._ = 0.12; Z_task−exp__._ = 11.44, *p* < 0.01) and emotional CE (rwc_task_ = 0.48, rwc_exp__._ = 0.38; Z_task−exp__._ = 4.16, *p* < 0.01), though we find no differences for cognitive CE.

#### Traditional marketing

Both traditional marketing strategies relate significantly to CE. Product performance relates more strongly to emotional CE (rwc = 0.60, *p* < 0.05) than to cognitive CE (rwc = 0.50, *p* < 0.05; Z_CCE−ECE_ = 9.28, *p* < 0.01) or behavioral CE (rwc = 0.47, *p* < 0.05; Z_BCE−ECE_ = 14.02, *p* < 0.01).^3^ Brand associations exhibit positive links to all three CE dimensions, with stronger effects for cognitive CE (rwc = 0.58, *p* < 0.05; Z_BCE−CCE_ = 19.59, *p* < 0.01) and emotional CE (rwc = 0.59, *p* < 0.05; Z_BCE−ECE_ = 17.55, *p* < 0.01) than for behavioral CE (rwc = 0.41, *p* < 0.05). When we compare the effect sizes of the CE-specific (task-based and experiential) initiatives with traditional marketing strategies (product performance and brand associations), we consistently find stronger effects sizes for the latter (all *p* < 0.01).

#### Consequences of CE

All CE dimensions relate to behavioral intentions, including behavioral CE (rwc = 0.49, *p* < 0.05), cognitive CE (rwc = 0.50, *p* < 0.05), and emotional CE (rwc = 0.55, *p* < 0.05). They also relate to behavioral outcomes: behavioral CE (rwc = 0.24, *p* < 0.05), cognitive CE (rwc = 0.21, *p* < 0.05), and emotional CE (rwc = 0.28, *p* < 0.05). In summary, firms benefit from engaging customers, and all CE dimensions matter.

The results of the effect size integration are robust to publication bias; the FSNs exceed the suggested tolerance levels (Rosenthal, 1979). The funnel plots do not indicate publication bias either. However, the significant Q-tests and wide credibility intervals suggest substantial variance in effect sizes and the need for moderator analyses. According to the power tests, our statistical analyses have sufficient power (> 0.5). The high shared variances (R^2^) and high BESDs also suggest that the antecedents we examine have great practical relevance for explaining CE, and CE is important for explaining marketing outcomes.

### Structural equation modeling results

We used structural equation modeling (SEM) to test the meta-analytic framework and mediating effects; Web Appendix G contains the correlation matrix we used for this SEM. The fit of the model is good, according to the results in Table 4.

**Table 4:** Testing the meta-analytic framework of CE

Relationship	Estimate	t-value
Antecedents		
CE marketing		
Task-based initiatives → Cognitive CE	0.18*	10.80
Task-based initiatives → Emotional CE	0.19*	14.00
Task-based initiatives → Behavioral CE	—	
Experiential initiatives → Cognitive CE	0.21*	12.84
Experiential initiatives → Emotional CE	0.09*	6.26
Experiential initiatives → Behavioral CE	—	
Traditional marketing		
Product performance → Cognitive CE	0.11*	5.66
Product performance → Emotional CE	0.19*	11.63
Product performance → Behavioral CE	0.17*	8.77
Brand associations → Cognitive CE	0.40*	23.36
Brand associations → Emotional CE	0.17*	10.92
Brand associations → Behavioral CE	—	
CE dimensions		
Cognitive CE → Emotional CE	0.39*	24.68
Cognitive CE → Behavioral CE	0.25*	12.08
Emotional CE → Behavioral CE	0.30*	13.07
Consequences		
Task-based initiatives → Behavioral intentions	0.25*	16.06
Experiential initiatives → Behavioral intentions	0.20*	12.71
Product performance → Behavioral intentions	0.05*	2.54
Brand associations → Behavioral intentions	0.26*	15.23
Cognitive CE → Behavioral intentions	—	
Emotional CE → Behavioral intentions	—	
Behavioral CE → Behavioral intentions	0.29*	17.66
Task-based initiatives → Behavioral outcome	—	
Experiential initiatives → Behavioral outcome	—	
Product performance → Behavioral outcome	—	
Brand associations → Behavioral outcome	0.28*	14.13
Emotional CE → Behavioral outcome	—	
Cognitive CE → Behavioral outcome	—	
Behavioral CE → Behavioral outcome	—	
Behavioral intentions → Behavioral outcome	0.26*	13.25
Model fit:		
Goodness-of-fit index	0.97	
Root mean residual	0.04	
Standardized mean residual	0.04	

This table displays only significant effects. A dash indicates a nonsignificant path. * *p* < 0.05

Regarding *CE marketing initiatives*, task-based (γ = 0.18, *p* < 0.05) and experiential (γ = 0.21, *p* < 0.05) initiatives relate to cognitive CE, with no difference between them (*p* > 0.05). For emotional CE, task-based (γ = 0.19, *p* < 0.05) and experiential (γ = 0.09, *p* < 0.05) initiatives exert positive effects, and the path coefficient is stronger for task-based than experiential initiatives (γ_task_ = 0.19 vs. γ_exper__._ = 0.09, t = 5.35, *p* < 0.01). Neither task-based nor experiential initiatives relate to behavioral CE (both *p* > 0.05).

With respect to *traditional marketing*, product performance (γ = 0.11, *p* < 0.05) and brand associations (γ = 0.40, *p* < 0.05) relate to cognitive CE, but when we combine them in the SEM, product performance loses relevance as driver of cognitive CE. Product performance displays weaker effects than task-based initiatives (γ_product_ = 0.11 vs. γ_task_ = 0.18, *t* = 2.54, *p* < 0.01) and experiential initiatives (γ_product_ = 0.11 vs. γ_exper__._ = 0.21, *t* = 3.90, *p* < 0.01), while the effects of brand associations are stronger than those of task-based initiatives (γ_brand_ = 0.40 vs. γ_task_ = 0.18, *t* = 9.55, *p* < 0.01) and experiential initiatives (γ_brand_ = 0.40 vs. γ_task_ = 0.21, *t* = 8.06, *p* < 0.01). For emotional CE, both product performance (γ = 0.19, *p* < 0.05) and brand associations (γ = 0.17, *p* < 0.05) exert positive effects. Whereas we find no difference in the effects of these traditional marketing strategies with task-based initiatives (both *p* > 0.05), the effects of product performance (γ_product_ = 0.19 vs. γ_exper__._ = 0.09, t = 4.78, *p* < 0.01) and brand associations (γ_brand_ = 0.17 vs. γ_exper__._ = 0.09, t = 3.95, *p* < 0.01) are stronger than those of experiential initiatives. Product performance has a significant effect (γ = 0.17, *p* < 0.05) on behavioral CE, but brand associations do not (*p* > 0.05).

Regarding the *CE dimensions*, emotional CE (β = 0.30, *p* < 0.05) and cognitive CE (β = 0.25, *p* < 0.05) relate to behavioral CE. We also uncover the expected link between cognitive CE and emotional CE (β = 0.39, *p* < 0.05).

Regarding the *consequences of CE*, none of the CE dimensions has a direct effect on behavioral outcomes (all *p* > 0.05). Instead, behavioral CE relates to behavioral intentions (β = 0.29, *p* < 0.05), which influences behavioral outcomes (β = 0.26, *p* < 0.05). In addition, task-based (γ = 0.25, *p* < 0.05) and experiential (γ = 0.20, *p* < 0.05) initiatives relate to behavioral intentions, but not to behavioral outcomes. Whereas product performance (γ = 0.05, *p* < 0.05) relates to behavioral intentions, brand associations relate to both behavioral intentions (γ = 0.26, *p* < 0.05) and behavioral outcomes (γ = 0.28, *p* < 0.05). Thus, the results suggest direct effects of different strategies on marketing outcomes and indirect effects through CE (Web Appendix H).

We acknowledge the possibility of reverse causality, such that behavioral CE might induce cognitive and emotional CE (Sussman & Gifford, 2019), such as through self-perception processes or misattribution of arousal. The results of testing this alternative model provide support for the proposed conceptualization (Web Appendix I).

### Moderator test results

In Table 5, we summarize the moderating influences of the four platform characteristics in terms of explaining when different CE initiatives improve CE and when increases in CE lead to more positive marketing outcomes. Although we only propose that two platform characteristics moderate CE formation, we test for the influence of all of them.

**Table 5:** Moderator analysis results

Relationship	k	Intercept	Intensity of interaction (1 = continuous; 0 = spot)	Richness of Interaction (1 = rich; 0 = lean)	Platform type (1 = digital; 0 = physical)	Initiator of interaction (1 = customer; 0 = firm)	Data collection (1 = single industry; 0 = multiple)	Research design (1 = survey; 0 = other)	Publication status (1 = publ.; 0 = unpubl.)	Study year	Publication quality
Antecedents											
CE marketing											
Task-based initiatives → Cognitive CE	15	0.44*	0.04	− 0.11	—	—	− 0.21	—	0.30	0.08*	− 0.01
Task-based initiatives → Emotional CE	18	0.48*	0.26*	0.05	—	—	− 0.29*	—	− 0.05	0.00	0.05
Task-based initiatives → Behavioral CE	48	0.08	0.02	− 0.12*	− 0.11	0.05	0.01	0.31*	0.02	− 0.01	0.06*
Experiential initiatives → Cognitive CE	11	0.38*	− 0.02	0.02	—	0.00	—	—	—	—	—
Experiential initiatives → Emotional CE	11	0.35*	0.04	0.13	—	− 0.07	—	—	—	—	—
Experiential initiatives → Behavioral CE	33	− 0.15	− 0.20*	− 0.03	0.09	0.41	0.05	0.35*	0.23*	− 0.02	
Traditional marketing											
Product performance → Cognitive CE	33	0.48*	− 0.11	− 0.02	− 0.02	0.03	0.06	0.00	0.06	0.00	− 0.01
Product performance → Emotional CE	25	0.53*	− 0.14	− 0.20	0.06	− 0.12	0.03	—	0.23	− 0.01	− 0.07
Product performance → Behavioral CE	60	0.09	− 0.08	− 0.12*	0.07	− 0.03	− 0.01	0.34*	0.06	− 0.01	− 0.01
Brand associations → Cognitive CE	31	0.70*	− 0.11	0.13	0.02	− 0.03	− 0.07	− 0.18	− 0.01	− 0.03	0.04
Brand associations → Emotional CE	23	0.67*	− 0.34*	− 0.16	0.18	0.18	− 0.02	− 0.12	0.10	− 0.02	− 0.02
Brand associations → Behavioral CE	63	0.13	0.07	0.15	0.03	− 0.06	0.07	0.32*	− 0.02	0.02	− 0.01
Consequences											
Cognitive CE → Behavioral intentions	56	0.60*	0.01	0.04	− 0.10	0.06	− 0.11*	− 0.04	0.01	− 0.01	0.01
Emotional CE → Behavioral intentions	54	0.60*	0.00	0.08*	− 0.14*	0.18*	− 0.13*	0.13*	0.01	0.01	− 0.03
Behavioral CE → Behavioral intentions	113	0.77*	0.04	0.03	− 0.16*	0.08*	− 0.06	− 0.04	− 0.02	0.01	− 0.05*
Cognitive CE → Behavioral outcome	16	0.25	− 0.17	0.14	—	− 0.09	− 0.03	0.12	0.01	—	—
Emotional CE → Behavioral outcome	14	0.07	− 0.26*	− 0.35*	—	—	0.10	0.31*	0.25*	—	—
Behavioral CE → Behavioral outcome	44	0.26	0.02	− 0.10	− 0.09	− 0.04	0.05	0.27*	0.02	0.02	0.00
			H1–H3	H4–H6	H7–H8	H9					

k = number of effect sizes. A dash indicates that a moderator could not be tested. * *p* < 0.05

#### Intensity of platform interaction

The moderating effects of the intensity of the interaction involve both the antecedents and consequences of CE dimensions. As predicted, the effects of task-based initiatives on emotional CE (H1; b = 0.26, *p* < 0.05) are greater for continuous than spot interactions. In line with our predictions, we observe weaker effects of experiential initiatives on behavioral CE for continuous interactions (H2; b = − 0.20, *p* < 0.05). The effect sizes of emotional CE on behavioral outcomes also diminish for continuous interactions (H3b; b = − 0.26, *p* < 0.05), but no differences arise for behavioral intentions, so we cannot confirm H3a.

#### Richness of platform interaction

This moderator is relevant for both the consequences and the antecedents of CE dimensions too. We find weaker effects of task-based initiatives on behavioral CE for rich interactions (b = − 0.12, *p* < 0.05), in line with our predictions in H5 but contrary to H4a. That is, our findings align with the task–media fit hypothesis. We do not find any moderating effect for the relationships of experiential-based initiatives with CE dimensions and thus must reject H4b. Among the consequences, the effect size of emotional CE on behavioral intentions is greater for rich interactions (b = 0.08, *p* < 0.05), contrary to H6a. Information provided in rich interactions seems to reassure customers and motivate them to maintain their relationship with a firm. In line with our predictions in H6b, emotional CE has weaker effects on behavioral outcomes in rich interactions (b = − 0.35, *p* < 0.05).

#### Platform type

This moderator seems more important for the consequences of CE than for its antecedents. As expected, we observe no differences between digital and physical platforms regarding the antecedents of the CE dimensions. But two significant effects emerge in this analysis, in line with our predictions: The effects of behavioral CE (b = − 0.16, *p* < 0.05) and emotional CE (b = − 0.14, *p* < 0.05) on behavioral intentions are weaker for digital platforms, in support of H8a but contrary to H7a. This finding is in line with customers’ negative views of digital versus physical interactions. Yet because we do not observe differences for behavioral outcomes, we cannot confirm H8b and H7b.

#### Initiator of platform interaction

For the initiator of platform interaction moderator, we again find no significant effects for antecedents of CE. As expected, the moderating effects are more pertinent to the consequences of CE. Behavioral CE displays stronger effects on behavioral intentions (b = 0.08, *p* < 0.05) if the customer initiates the interaction, and the results are similar for emotional CE (b = 0.18, *p* < 0.05), in support of H9a. But in contrast with H9b, we do not find differences for behavioral outcomes.

#### Traditional marketing strategies

In additional post hoc analyses, we test for the moderating effects of platform characteristics on traditional marketing strategies. Regarding intensity, we observe weaker effects of brand associations on emotional CE (b = − 0.34, *p* < 0.05), in contrast with our predictions; that is, brand associations appear are more effective in spot interactions. We find no differences for product performance. For richness, we find weaker effects of product performance on behavioral CE in rich interactions (b = − 0.12, *p* < 0.05), in line with our predictions. No difference emerges for brand associations. As expected, no influences result from the platform type or initiator of the platform interaction.

#### Control variables

The results of the moderator tests remain consistent when we control for method moderators. For a few relationships, the effect sizes are weaker in single- compared with multi-industry studies, and some relationships appear stronger if the studies rely on surveys. The results are in line with the subgroup analysis (Web Appendix J).

## Discussion

With the general assumption that their firm can benefit from engaging customers, managers invest in CE marketing, using different engagement strategies deployed on various engagement platforms. With a meta-analysis and data from 395 samples, involving 434,233 customers, we develop and test a unifying framework to help them optimize these investments. In particular, the results give managers insights into which strategies improve CE and when greater CE prompts more positive marketing outcomes.

### When do task-based, experiential, or traditional marketing initiatives improve CE?

Both task-based and experiential initiatives relate positively to cognitive and emotional CE, but the effects on behavioral CE are indirect, through other CE dimensions. Experiential initiatives tend to be less effective in driving cognitive and behavioral CE than are task-based initiatives (Tables 3 and 4), likely because triggering self-transformation processes through experiential initiatives is inherently difficult. Furthermore, experiential initiatives are less effective than traditional marketing strategies in driving emotional CE, but task-based initiatives exhibit similar effectiveness. Both CE-specific initiatives drive cognitive CE better than product performance does, but they are not as effective as brand associations. Thus, strategies that deliberately stimulate CE can be as effective as strategies that rely on organic inducements of engagement.

By incorporating insights from platform theory, we also can detail which platform characteristics determine the effectiveness of different engagement strategies. Through this investigation of the moderating effects of the intensity of interaction, richness, platform types, and initiator, we establish contextual explanations for when engagement strategies are more effective for driving CE. Thus, we provide a general overview of the distinctly moderated relationships between engagement strategies and CE dimensions.

First, the intensity and richness of the platform interactions are relevant, whereas we do not find moderating effects of platform type and the initiator on the formation of CE. The platform and the initiator do not function as contextual differentiators, unlike the platform’s underlying traits. The lack of a moderating effect of the platform type might reflect the widespread use of digital platforms for engagement initiatives, such that many firms already use multiple channels to engage customers (Meire et al., 2019). Notably, both interaction intensity and richness exert mixed influences across emotional and behavioral CE.

Second, engagement strategies might be less susceptible to moderation by platform traits. But when the moderation effects emerge, they have divergent implications for different strategies. For example, continuous interactions strengthen the relationship of task-based initiatives with emotional CE, but they weaken the relationship of experiential initiatives with behavioral CE. The former strengthening effect implies that customers perform better on tasks when they interact more with the firm. The latter effect resonates with predictions of wear-out effects; experiential initiatives become less effective over time, because unusual events that increase emotional intensity cannot be redundant or frequent. Our finding that the effects of task-based initiatives on behavioral CE are weaker during rich interactions is in line with the task–media fit hypothesis, which suggests that structured tasks require lean media.

Third, the importance of traditional marketing strategies varies with the intensity and richness of the platform interaction. Even if product performance and brand association both rely on the activation of memories to influence CE, the triggers may differ. Specifically, product performance is more important for driving behavioral CE in lean than in rich interactions; lean interactions help customers focus on specific cues that activate their previous positive product experiences. Brand associations are more important for driving emotional CE in spot interactions than continuous interactions; it seems that wear-out effects reduce customer attention and recall.

### When does greater CE evoke more positive marketing outcomes?

The meta-analysis results suggest that, on average, firms benefit from engaging customers. The CE dimensions influence unique marketing outcomes, and we can establish pathways to behavioral outcomes, through intentions. In addition, emotional and cognitive CE exert indirect effects through behavioral CE. Failing to acknowledge the mediating effects can lead to underestimates of the effectiveness of CE as a predictor of behavioral outcomes. This finding may help explain the mixed results in prior studies regarding the effects of the CE dimensions on various outcome variables.

Regarding the moderators we test, we note some important differences in the results related to the formation of CE and its translation into firm outcomes. The intensity of the platform interaction is mainly relevant for the formation of CE, whereas the platform type and initiator are mainly relevant for translating CE into marketing outcomes. The richness of the interaction seems more broadly influential, in that it moderates both the formation and translational pathways. Specific to the links between CE dimensions and marketing outcomes, we need to consider additional moderating effects, related to the platforms’ type and traits. In some conditions, behavioral CE even can function as a sole predictor of marketing outcomes; in others, emotional CE exerts direct effects. For example, emotional CE is more important in driving marketing outcomes during customer-initiated interactions but twice less important in continuous or rich interactions.

In seeking to establish such moderating effects, we identify and define some that have not been tested before. For example, behavioral and emotional CE display weaker effects on behavioral intentions for digital than physical platforms; their effects also are weaker for firm- than customer-initiated interactions. Negative views of digital versus real-world exchanges undermine the effectiveness of engagement for driving marketing outcomes in digital environments, but in customer-initiated interactions, CE has greater personal relevance and thus more powerful effects. We find that emotional CE exhibits weaker effects on behavioral outcomes for continuous than spot interactions and for rich than lean interactions. Because spot interactions are constrained in time and topic, the engagement object is more salient in such interactions, enhancing its personal relevance. Customer decision-making is more complex in rich interactions, so engagement also loses relevance as a predictor of marketing outcomes, because it is just one of many cues to consider. Finally, cognitive CE displays similar effectiveness across different platforms. Overall, these insights highlight the usefulness of integrating platform theory with CE marketing theory. Table 6 summarizes the results of our hypotheses testing.

**Table 6:** Summary of hypotheses test results

Effect	Finding	Conclusion
Intensity of Platform Interaction		
H1: The positive relationships of task-based initiatives with CE dimensions are stronger for continuous than for spot interactions.	H1 (emotional CE: ↑)	Continuous interactions strengthen the relationship of task-based initiatives with emotional CE. This strengthening effect implies that customers perform better on tasks when they interact more with the firm. No differences were observed for behavioral and cognitive CE.
H2: The positive relationships of experiential initiatives with CE dimensions are weaker for continuous than for spot interactions.	H2 (behavioral CE: ↓)	Continuous interactions weaken the relationship of experiential initiatives with behavioral CE. This effect resonates with predictions of wear-out effects; experiential initiatives become less effective over time, because unusual events that increase emotional intensity cannot be redundant or frequent. No differences were observed for emotional and cognitive CE.
H3: The positive relationships of CE dimensions with (a) behavioral intentions and (b) behavioral outcomes are weaker for continuous than for spot interactions.	H3a (ns) H3b (emotional CE: ↓)	Emotional CE exhibits weaker effects on behavioral outcomes for continuous than spot interactions. Because spot interactions are time- and issue-limited, the focal issue is more salient in such interactions, enhancing personal relevance. No differences were observed for cognitive and behavioral CE, suggesting that salience effects only apply to emotional CE. Also, no differences were observed for behavioral intentions.
Richness of platform interaction		
H4: The positive relationships of (a) task-based initiatives and (b) experiential-based initiatives with CE dimensions are stronger for rich than for lean interactions. H5: The positive relationships of task-based initiatives with CE dimensions are weaker for rich than for lean interactions.	H4a (behavioral CE: ↓) H4b (ns) H5 (behavioral CE: ↓)	The effects of task-based initiatives on behavioral CE are weaker during rich interactions, in line with the task–media fit hypothesis. Experiential initiatives have the same effectiveness across rich and lean platforms. It seems that self-transformation processes associated with experiential initiatives do not rely on rich platform interactions; lean interactions can also support self-transformation processes. No differences were observed for cognitive and emotional CE.
H6: The positive relationships of CE dimensions with (a) behavioral intentions and (b) behavioral outcomes are weaker for rich than for lean interactions.	H6a (emotional CE: ↑) H6b (emotional CE: ↓)	Emotional CE exhibits weaker effects on behavioral outcomes for rich than lean interactions. Customer decision-making is more complex in rich interactions, so engagement loses relevance as a predictor of marketing outcomes, because it is just one of many cues to consider. The effect on behavioral intentions is contrary to our predictions, suggesting that some moderating effects vary by outcome variable. No differences were observed for cognitive and behavioral CE.
Platform type		
H7: The positive relationships of CE dimensions with (a) behavioral intentions and (b) behavioral outcomes are stronger for digital than for physical platforms. H8: The positive relationships of CE dimensions with (a) behavioral intentions and (b) behavioral outcomes are weaker for digital than for physical platforms.	H7a (behavioral CE: ↓; emotional CE: ↓) H7b (ns) H8a (behavioral CE: ↓; emotional CE: ↓) H8b (ns)	Behavioral and emotional CE display weaker effects on behavioral intentions for digital than physical platforms. This finding is in line with customers’ negative views of digital versus physical interactions, which appear to undermine the effectiveness of engagement for driving marketing outcomes. No differences were observed for cognitive CE.
Initiator of platform interaction		
H9: The positive relationships of CE dimensions with (a) behavioral intentions and (b) behavioral outcomes are stronger for customer- than firm-initiated interactions.	H9a (behavioral CE: ↑; emotional CE: ↑) H9b (ns)	Behavioral and emotional CE display stronger effects for customer- than firm-initiated interactions. In customer-initiated interactions, CE has greater personal relevance and thus more powerful effects on outcomes. No differences were observed for cognitive CE.

↑= moderation that strengthens the proposed relationship; ↓ = moderation that weakens the proposed relationship

### Managerial implications

We summarize the implications of our findings for managers investing in CE marketing in Table 7. Our results can guide managers when they must choose between the two engagement strategies and when investing their CE marketing budgets across platforms in ways that increase the chances that their firm will benefit from engaging customers.

**Table 7:** Managerial implications

Issue	Implication
Which engagement initiative is most effective in driving CE?	
Task-based vs. experiential initiatives	Task-based initiatives are more effective than experiential initiatives, so managers should prioritize them. Managers also should realize the difficulty of triggering self-transformation in customers and thus exhibit caution when launching experiential initiatives.
Engagement marketing vs. traditional marketing	Both CE-specific marketing and traditional marketing can stimulate CE, though differences arise with the different CE dimensions. Thus, the approaches may be complementary, and managers should select them accordingly.
When should firms prioritize different engagement strategies?	
Continuous vs. spot interactions	Task-based initiatives are more effective on platforms that support continuous interactions; experiential initiatives gain importance on platforms that support spot interactions. Managers should assess whether the available platforms support spot or continuous interactions with customers.
Rich vs. lean interactions	Task-based initiatives are more effective on platforms that support lean rather than rich interactions. Managers should assess different platforms in terms of available response tools (e.g., sharing, liking, commenting) and message tools (e.g., video, audio, high-quality pictures).
Digital vs. physical platforms	No effects arise; managers can employ either kind of initiative on digital or physical platforms.
Customer- vs. firm-initiated interactions	No effects arise; managers can employ either kind of initiative in relation to customer- or firm-initiated interactions.
Do firms benefit from engaging customers?	
CE dimensions effects on marketing outcomes	On average, firms benefit from engaging customers. The three CE dimensions relate to actual purchase behaviors and intentions, though emotional and cognitive CE display indirect effects through behavioral CE. Thus, managers need to measure and monitor all CE dimensions.
When is CE likely to translate into marketing outcomes?	
Continuous vs. spot interactions	Emotional CE displays weaker effects on behavioral outcomes for continuous than spot interactions.
Rich vs. lean interactions	Emotional CE displays weaker effects on behavioral outcomes for rich than lean interactions.
Digital vs. physical platforms	Behavioral and emotional CE display weaker effects on behavioral intentions for digital than physical platforms.
Customer- vs. firm-initiated interactions	The effects of behavioral and emotional CE are stronger for customer- than firm-initiated interactions. Managers should enable instant feedback and live interaction on different platforms to encourage customer-initiated interactions or choose among platforms accordingly.

First, managers should realize that task-based initiatives, on average, are more effective than experiential initiatives and acknowledge the difficulty of triggering self-transformation in customers, especially compared with the relative ease of inducing ownership feelings through task-based initiatives. Firms can allocate their resources across engagement strategies that deliberately seek to motivate customers to invest resources and engage with the firm and traditional marketing strategies that organically lead to engagement. Both can stimulate CE, though differently across CE dimensions. In this sense, the approaches can complement each other, and managers should leverage them accordingly.

Second, regarding distinct conditions in which to prioritize different engagement strategies, we recommend that managers map their engagement strategies onto the characteristics of the platforms they are considering, using the matrix displayed in Table 1. By doing so, they can derive tactical marketing and communication plans, as well as align their budget allocations across different engagement platforms. In general, managers should use task-based initiatives on platforms that support continuous interactions, because they facilitate customer learning (see the platforms in columns A and B in Table 1). Task-based initiatives also are more effective on platforms that support lean interactions rather than those that feature distracting and unnecessary information (columns B and D). Thus, managers have to be more careful when choosing rich engagement platforms, such as social games and social worlds, for task-based initiatives (e.g., Facebook’s metaverse; Kaplan & Haenlein, 2010). If managers instead seek to encourage customers’ self-transformations, they should install experiential initiatives on platforms that support spot interactions, during which customers have not yet fully reflected on the firm or its brand (columns C and D). No differences were observed for the other two moderators, so managers can employ either kind of initiative on digital or physical platforms. They can also employ either kind of initiative in relation to customer- or firm-initiated interactions.

Third, on average, firms benefit from engaging customers. The three CE dimensions relate to behavioral outcomes and intentions, though emotional and cognitive CE display indirect effects through behavioral CE. Thus, practitioners should continuously measure and monitor their effects. By applying CE funnels or conversion tables, they can determine the percentage of customers who are engaged on each CE dimension.

Fourth, managers should use all the classification criteria we include in our meta-analysis when assessing returns on CE efforts: intensity, richness, platform type, and interaction initiator. They generally can expect stronger effects of the CE dimensions on marketing outcomes with spot interactions (columns C and D, Table 1), on physical platforms (rows 3 and 4), and for customer-initiated interactions (rows 2 and 4). The effects for rich versus lean interactions vary by outcome variable. Thus, managers should carefully map the three CE dimensions on platform characteristics to choose the most effective platform in terms of translating CE into their desired marketing outcomes.

### Research agenda

In presenting several avenues for further research, we emphasize issues related to the effectiveness of engagement strategies, firm benefits of engaging customers, and method- and data-related issues (Table 8).

**Table 8:** Research agenda

Issues	Exemplary Research Directions
Effectiveness of engagement strategies	
Underlying mechanism	• Assess the mediating effects of psychological ownership and self-transformation; extant studies measure these mechanisms only infrequently despite their central role in CE marketing theory
Types of engagement strategies	• Assess the effectiveness of different types of task-based initiatives (e.g., financial rewards vs. social recognition) and experiential initiatives (e.g., crowdsourcing events vs. brand fests)
Interplay with traditional marketing	• Examine the interplay of CE initiatives and traditional marketing strategies (e.g., idea competition accompanied by a branding campaign); examine how to combine both strategies to increase their effectiveness
Continuous vs. spot interactions	• Design experiential initiatives to increase effectiveness in continuous interactions and task-based initiatives in spot interactions (e.g., explore how to support customer learning in spot interactions)
Rich vs. lean interactions	• Explore when task-based initiatives benefit from rich media and when experiential initiatives benefit from lean media
Digital vs. physical platforms	• Explore types of task-based and experiential strategies that work better on digital platforms than physical platforms, and vice versa. New technologies (e.g., augmented reality) may enhance the effectiveness of certain experiential initiatives on digital platforms
Customer- vs. firm-initiated interactions	• Explore whether engagement strategies differ in effectiveness depending on the specific customer motivation to initiate the interaction; prior literature points toward different customer motivations to initiate an interaction
Further context differences	• Explore the effectiveness of engagement strategies in multichannel environments (e.g., mixed digital and physical platforms), when customers engage with a focal firm across platforms
Firm benefits of engaging customers	
CE conceptualization	• Use models and theories that go beyond a dual emotion–cognition process; use insights from consumer psychology to examine the processes by which cognitive CE is altered by emotions and emotional CE by cognitions
Performance outcomes	• Examine other outcome variables of CE at the firm level (e.g., sales, profitability), instead of the customer level (behavioral intentions and behaviors); consider firm profitability as an outcome (return on investment)
Continuous vs. spot interactions	• Check for contexts in which effectiveness of cognitive and behavioral CE depends on the intensity of the interaction; research on episodic versus continuous engagement might provide useful insights, for example
Rich vs. lean interactions	• Consider different types of richness (e.g., content, format) and their differential effects on effectiveness of CE; use media richness theories to identify other characteristics that describe the platform interaction
Digital vs. physical platforms	• Explore whether segment-specific preferences exist regarding digital versus physical platforms, including generational differences (e.g., Millennials, Generation Z) but other distinctions of customers too
Customer- vs. firm-initiated interactions	• Explore how firms should initiate interactions to increase their personal relevance
Further context differences	• Assess the CE marketing effectiveness of emerging technologies (e.g., virtual reality, augmented reality, social robots) to address their unique characteristics
Method- and data-related issues	
Longitudinal research	• Adopt longitudinal research designs to examine the entire CE process; the development of short- or long-term engagement is difficult to assess with cross-sectional data
Experimental research	• Use experiments to check for reverse causality (behavior → cognition, emotion) and test the processes suggested by consumer psychology and marketing research (e.g., emotion regulation)
Qualitative research	• Use qualitative approaches (e.g., in-depth interviews, focus groups) to explore underlying reasons for some of the surprising findings of the meta-analysis

First, more detailed research is needed to gauge the effectiveness of engagement strategies, including measures of the specific mechanism (e.g., psychological ownership, self-transformation) by which experiential and task-based initiatives relate to CE dimensions. Scholars also might differentiate types of task-based initiatives (e.g., financial rewards vs. social recognition) or experiential initiatives (e.g., crowdsourcing events vs. brand fests), then assess their distinct interactions with traditional marketing strategies (e.g., idea competition accompanied by a branding campaign). Combining both sets of strategies may enhance their effectiveness. Regarding platform differences, we also suggest extended research into platform characteristics. One research objective might be to explore how to design task-based initiatives that are effective in spot interactions. In some cases, task-based initiatives might benefit from rich media, and experiential initiatives can benefit from lean media. Perhaps specific types of task-based and experiential strategies work better on digital platforms than physical platforms, or vice versa. Certain engagement strategies also might become effective, depending on the customer’s specific motivation for initiating an interaction. Beyond deepening understanding of the platform characteristics that we study, scholars could consider other moderators too, such as multichannel environments (mixed digital–physical platforms), in which customers can engage with the same firm across multiple platforms.

Second, we call for more research into the benefits for firms, across more marketing outcomes. Alternative outcome variables of CE might pertain to the firm level (e.g., sales, profitability), instead of the customer level. Continued studies also should consider the costs of engagement strategies to gauge firm profitability (return on investment). Furthermore, we conceptualize CE as a three-dimension, multivariate construct; further research on emotion–cognition interactions could provide more nuanced, less dichotomous views on these CE dimensions (Pessoa, 2018), as supported by the theories outlined in Web Appendix K. For example, a process model of emotion regulation suggests that people regulate their emotions through four underlying processes (situation selection, situation modification, attentional deployment, and cognitive change; Gross, 1998). Regarding platform differences, scholars could build on our framework to test for potential segment-specific preferences for digital or physical platforms, such as generational differences (e.g., Generation Z). Another interesting question involves how firms might initiate interactions to increase their personal relevance to customers, which should make CE more influential. In in-depth considerations of different types of richness (e.g., content, format) and their effects on CE effectiveness, media richness literature also might be useful. Finally, we encourage scholars to identify other factors that might influence the effectiveness and impacts of engagement strategies on marketing outcomes. For example, emerging technologies (e.g., virtual reality, social robots) and their unique characteristics likely have distinct effects.

Third, CE marketing literature would benefit from more diverse methodologies. Most existing studies use cross-sectional data, but marketing strategies can lose effectiveness over time, due to wear-out effects. Longitudinal research designs can specify the relevance of such strategies over time. In addition, we find that emotional and cognitive CE relate to behavioral CE. By moving beyond the widespread use of surveys, researchers conducting experiments could check further for reverse causality (i.e., can behavior induce emotional and cognitive bonds?). More qualitative approaches also might help reveal the reasons for some of our surprising findings. We hope this agenda for advancing CE research proves inspiring.

## Supplementary information

Below is the link to the electronic supplementary material.

ESM 1 (DOCX 566 KB)

## Footnotes

1 For a more detailed comparison of the contributions of the current meta-analysis, relative to these prior efforts, please see Web Appendix A.

2 Psychological ownership and self-transformation are suggested as mediating mechanisms to explain the relationship between CE marketing strategies and CE (Harmeling et al., 2017). They are not seen as components of CE but as underlying mechanisms to explain the relationship. Because most empirical studies do not measure these mediators, we do not include them in the meta-analysis.

3 The higher-order conceptualization shares the most variance with emotional CE (rwc = 0.84; R^2^ = 71%), followed by cognitive (rwc = 0.75; R^2^ = 56%) and behavioral (rwc = 0.63; R^2^ = 40%) CE. A similar observation applies to correlations with the unidimensional version: emotional CE (rwc = 0.84; R^2^ = 71%) is stronger than cognitive (rwc = 0.75; R^2^ = 56%) or behavioral (rwc = 0.62; R^2^ = 38%) CE.
