# Supplementary material for: Effectiveness of engagement initiatives across engagement platforms: A meta-analysis
Source: J Acad Mark Sci. 2023 Feb 28:1–25. Online ahead of print. doi: 10.1007/s11747-023-00925-7 (PMC9972333; doi:10.1007/s11747-023-00925-7)
Supplement: Supplementary file 1 — (DOCX 566 KB) [file 11747_2023_925_MOESM1_ESM.docx]

**Effectiveness of Engagement Initiatives Across Engagement Platforms:**

**A Meta-Analysis**

**WEB APPENDIX**

| Web Appendix A: Comparison of the Present Meta-Analysis with Related CE Studies 2 |
| --- |
| Web Appendix B: Definitions of CE 3 |
| Web Appendix C: Testing Other CE Antecedents 7 |
| Web Appendix D: Method Appendix 8 |
| Web Appendix E: Measurement Based in Theory 10 |
| Web Appendix F: Testing Alternative CE Conceptualizations 14 |
| Web Appendix G: Correlation Matrix 17 |
| Web Appendix H: Testing Indirect Effects of Antecedents through CE dimensions 18  Web Appendix I: Testing Reverse Causality of CE Dimensions in Meta-Analytic Framework 19 |
| Web Appendix J: Results of Subgroup Analysis 20 |
| Web Appendix K: Avenues for Research on CE Construct 22 |

**Web Appendix A: Comparison of the Present Meta-Analysis with Related CE Studies**

|  | **Present**  **Study** | **Harmeling et al. (2017)** | **Santini et al. (2020)** |
| --- | --- | --- | --- |
| **Method** | Meta-analysis | Conceptual and quasi-experiment | Meta-analysis |
| **Type of CE** | Multidimensional: cognitive, emotional, behavioral considering interrelationships | Unidimensional: behavioral | Two-dimensional: psychological state, behavioral |
| **Data** | N = 434,233  (395 samples) | N = 1,203 | N = 161,059  (92 samples) |
|  |  |  |  |
| **Antecedents** |  |  |  |
| Task-based initiatives → Cognitive CE | ✓ | 🗶 | 🗶 |
| Task-based initiatives → Emotional CE | ✓ | 🗶 | 🗶 |
| Task-based initiatives → Behavioral CE | ✓ | ✓ (via psych. ownership; not tested empirically) | 🗶 |
| Experiential initiatives → Cognitive CE | ✓ | 🗶 | 🗶 |
| Experiential initiatives → Emotional CE | ✓ | 🗶 | 🗶 |
| Experiential initiatives → Behavioral CE | ✓ | ✓ (via self-transform.) | 🗶 |
| Product performance → Cognitive CE | ✓ | 🗶 | 🗶 |
| Product performance → Emotional CE | ✓ | 🗶 | 🗶 |
| Product performance → Behavioral CE | ✓ | ✓ (not tested empirically) | 🗶 |
| Brand associations → Cognitive CE | ✓ | 🗶 | 🗶 |
| Brand associations → Emotional CE | ✓ | 🗶 | 🗶 |
| Brand associations → Behavioral CE | ✓ | ✓ (not tested empirically) | 🗶 |
|  |  |  |  |
| **CE Dimensions** |  |  |  |
| Cognitive CE → Emotional CE | ✓ | 🗶 | 🗶 |
| Cognitive CE → Behavioral CE | ✓ | 🗶 | 🗶 (psychological state) |
| Emotional CE → Behavioral CE | ✓ | 🗶 | 🗶 (psychological state) |
|  |  |  |  |
| **Consequences** |  |  |  |
| Cognitive CE → Behavioral intentions | ✓ | 🗶 | 🗶 (psychological state) |
| Emotional CE → Behavioral intentions | ✓ | 🗶 | 🗶 (psychological state) |
| Behavioral CE → Behavioral intentions | ✓ | 🗶 | 🗶 (not modeled) |
| Cognitive CE → Behavioral outcome | ✓ | 🗶 | 🗶 |
| Emotional CE → Behavioral outcome | ✓ | 🗶 | 🗶 |
| Behavioral CE → Behavioral outcome | ✓ | 🗶 | 🗶 |
| Behav. intentions → Behavioral outcome | ✓ | 🗶 | 🗶 |
|  |  |  |  |
| **Platform Moderators** |  |  |  |
| Intensity of platform interaction | ✓ | 🗶 | 🗶 |
| Richness of platform interaction | ✓ | 🗶 | 🗶 |
| Platform type | ✓ | 🗶 | 🗶 (3 social media types) |
| Initiator of platform interaction | ✓ | 🗶 | 🗶 |

**Web Appendix B: Definitions of CE**

| **Author(s)** | **Definition** | **Construct** | **Object** | **Dimensionality** | **Study type** | **Assessment** |
| --- | --- | --- | --- | --- | --- | --- |
| Beckers et al. (2019) | “[B]ehaviors that go beyond customer transactions, such as, for example, word-of-mouth (e.g., van Doorn et al. 2010). This means that we focus on customer engagement behavior, taking the perspective of the value-creating role of the customer” (p. 367). | Customer engagement behavior | Firm | Unidimensional: behavioral | Empirical | Does not consider emotional and cognitive CE |
| Mirbagheri and Najmi (2019) | “We define consumers’ engagement with a social media activation campaign as the extent of cognitive, affective, and behavioral energies that consumers simultaneously and holistically devote into a campaign” (p.380). | Customer engagement | Social media campaigns | Second-order: cognitive, emotional, and behavioral | Empirical | Suggests a second-order construct rather than interrelated lower-order constructs |
| Dessart (2017) | “The state that reflects consumers’ positive individual dispositions towards the community and the focal brand as expressed through varying levels of affective, cognitive and behavioral manifestations that go beyond exchange situations” (p. 377). | Customer brand engagement | Brand, community | Second-order: cognitive, emotional, and behavioral | Empirical | Suggests a second-order construct rather than interrelated lower-order constructs |
| Harmeling et al. (2017) | “We therefore define customer engagement as a customer’s voluntary resource contribution to a firm’s marketing function, going beyond financial patronage” (p. 316). | Customer engagement | Firm | Unidimensional: behavioral | Empirical | Does not consider emotional and cognitive CE |
| Kumar and Pansari (2016) | “The attitude, behavior, the level of connectedness (1) among customers, (2) between customers and employees, and (3) of customers and employees within a firm” (p. 498). | Customer engagement | Firm | Unidimensional: behavioral | Empirical | Does not consider emotional and cognitive CE |
| Hollebeek et al. (2014) | “A consumer's positively valenced brand-related cognitive, emotional and behavioral activity during, or related to focal consumer/brand interactions” (p. 151). | Customer brand engagement | Brand | Multidimensional: cognitive, emotional, and behavioral | Empirical | Does not consider the interrelationships among CE dimensions |
| Jaakkola and Alexander (2014) | “Customers make voluntary resource contributions that have a brand or firm focus but go beyond what is fundamental to transactions, occur in interactions between the focal object and/or other actors, and result from motivational drivers” (p. 248). | Customer engagement behavior | Brand, firm | Unidimensional: behavioral | Conceptual | Does not consider emotional and cognitive CE |
| Vivek et al. (2014) | “CE goes beyond purchase and is the level of the customer’s (or potential customer’s) interactions and connections with the brand or firm’s offerings or activities, often involving others in the social network created around the brand/offering/activity” (p. 406). | Customer engagement | Brand | Multidimensional: cognitive, emotional, behavioral | Empirical | Does not consider the interrelationships among CE dimensions |
| Brodie et al. (2013) | “Consumer engagement in a virtual brand community involves specific interactive experiences between consumers and the brand, and/or other members of the community. Consumer engagement is a context-dependent, psychological state characterized by fluctuating intensity levels that occur within dynamic, iterative engagement processes. Consumer engagement is a multidimensional concept comprising cognitive, emotional, and/or behavioral dimensions, and plays a central role in the process of relational exchange where other relational concepts are engagement antecedents and/or consequences in iterative engagement processes within the brand community” (p. 107). | Customer engagement | Brand, community | Multidimensional: cognitive, emotional, and behavioral | Empirical | Does not consider the interrelationships among CE dimensions |
| Vivek et al. (2012) | CE is the intensity of an individual's participation in and connection with an organization's offerings or organizational activities, which either the customer or the organization initiates (p. 133). | Customer engagement | Firm | Multidimensional: cognitive, emotional, behavioral, and socially | Conceptual | Does not consider the interrelationships among CE dimensions |
| Brodie et al. (2011) | “Psychological state that occurs by virtue of interactive, cocreative customer experiences with a focal agent/object (e.g., a brand) in focal service relationships. It occurs under a specific set of context dependent conditions generating differing CE levels; and exists as a dynamic, iterative process within service relationships that cocreate value. CE plays a central role in a nomological network governing service relationships in which other relational concepts (e.g., involvement, loyalty) are antecedents and/or consequences in iterative CE processes. It is a multidimensional concept subject to a context- and/or stakeholder-specific expression of relevant cognitive, emotional and/or behavioral dimensions” (p.260). | Customer engagement | Brand | Multidimensional: cognitive, emotional, and behavioral | Conceptual | Does not consider the interrelationships among CE dimensions |
| Hollebeek (2011) | Customer brand engagement is defined as the level of an individual customer’s motivational, brand-related, and context-dependent state of mind, characterized by specific levels of cognitive, emotional, and behavioral activity in direct brand interactions (p. 790). | Customer brand engagement | Brand | Second-order: cognitive, emotional, and behavioral | Conceptual | Suggests a second-order construct rather than interrelated lower-order constructs |
| van Doorn et al. (2010) | “Customers’ behavioral manifestation toward a brand or firm, beyond purchase, resulting from motivational drivers (e.g., word-of mouth activity, recommendations, helping other customers, blogging, writing reviews and engaging in legal action” (p.253). | Customer engagement behavior | Brand, firm | Unidimensional: behavioral | Conceptual | Does not consider the interrelationships among CE dimensions |
| Sprott et al. (2009) | “An individual difference representing consumers’ propensity to include important brands as part of how they view themselves” (p. 92). | Brand engagement | Brand | Unidimensional | Empirical | CE dimensions are treated as reflective items of a unidimensional construct; differences between dimensions are not considered |
| Algesheimer et al. (2005) | “The consumer’s intrinsic motivation to interact and cooperate with community members” (p. 21). | Brand community engagement | Brand community | Unidimensional | Empirical | CE dimensions are treated as reflective items of a unidimensional construct; differences between dimensions are not considered |

**References**

Algesheimer, R., Dholakia, U. M., and Herrmann, A. (2005), “The social influence of brand community: Evidence from European car clubs,” *Journal of Marketing, 69*(3), 19-34.

Beckers, S. F., Van Doorn, J., and Verhoef, P. C. (2018), “Good, better, engaged? The effect of company-initiated customer engagement behavior on shareholder value,” *Journal of the Academy of Marketing Science*, 46(3), 366-383.

Brodie, R. J., Hollebeek, L. D., Jurić, B., and Ilić, A. (2011), “Customer engagement: Conceptual domain, fundamental propositions, and implications for research,” *Journal of Service Research, 14*(3), 252-271.

Brodie, R. J., Ilic, A., Juric, B., and Hollebeek, L. (2013), “Consumer engagement in a virtual brand community: An exploratory analysis,” *Journal of Business Research*, *66*(1), 105-114.

Dessart, L. (2017), “Social media engagement: A model of antecedents and relational outcomes,” *Journal of Marketing Management, 33*(5-6), 375-399.

Harmeling, C. M., Moffett, J. W., Arnold, M. J., and Carlson, B. D. (2017), “Toward a theory of customer engagement marketing,” *Journal of the Academy of Marketing Science*, 45(3), 312-335.

Hollebeek, L. D., Glynn, M. S., and Brodie, R. J. (2014), “Consumer brand engagement in social media: Conceptualization, scale development and validation,” *Journal of Interactive Marketing, 28*(2), 149-165.

Hollebeek, L. D. (2011), “Demystifying customer brand engagement: Exploring the loyalty nexus,” *Journal of Marketing Management*, 27(7-8), 785-807.

Jaakkola, E., and Alexander, M. (2014), “The role of customer engagement behavior in value co-creation: A service system perspective,” *Journal of Service Research*, 17(3), 247-261.

Kumar, V., and Pansari, A. (2016), “Competitive advantage through engagement,” *Journal of Marketing Research, 53*(4), 497-514.

Mirbagheri, S., and Najmi, M. (2019), “Consumers’ engagement with social media activation campaigns: Construct conceptualization and scale development,” *Psychology & Marketing*, *36*(4), 376-394.

Sprott, D., Czellar, S., and Spangenberg, E. (2009), “The importance of a general measure of brand engagement on market behavior: Development and validation of a scale,” *Journal of Marketing Research, 46*(1), 92-104.

Van Doorn, J., Lemon, K. N., Mittal, V., Nass, S., Pick, D., Pirner, P., and Verhoef, P. C. (2010), “Customer engagement behavior: Theoretical foundations and research directions,” *Journal of Service Research, 13*(3), 253-266.

Vivek, S. D., Beatty, S. E., Dalela, V., and Morgan, R. M. (2014), “A generalized multidimensional scale for measuring customer engagement,” *Journal of Marketing Theory and Practice*, 22(4), 401-420.

Vivek, S. D., Beatty, S. E., and Morgan, R. M. (2012), “Customer engagement: Exploring customer relationships beyond purchase,” *Journal of Marketing Theory and Practice*, 20(2), 122-146.

**Web Appendix C: Testing Other CE Antecedents**

| **Relationship** | **k** | **N** | **rwc** | **R^2^** | **BESD** | **CI_low_** | **CI_high_** | **CR_low_** | **CR_high_** | **Q** | **FSN** | **Power** |
| --- | --- | --- | --- | --- | --- | --- | --- | --- | --- | --- | --- | --- |
| Costs → Cognitive CE | 16 | 3749 | .11* | 1% | 1.25 | .04 | .18 | -.05 | .26 | 53* | 26 | >.999 |
| Costs → Emotional CE | 16 | 3749 | .22* | 5% | 1.56 | .08 | .36 | -.12 | .57 | 193* | 287 | >.999 |
| Costs → Behavioral CE | 27 | 7263 | .20* | 4% | 1.50 | .11 | .30 | -.11 | .51 | 312* | 1106 | >.999 |
| Costs → Unidimensional CE | 5 | 1969 | .24 | 6% | 1.63 | -.05 | .53 | -.18 | .66 | 154* | — | >.999 |
| Costs → Higher-order CE | 8 | 821 | .01 | 0% | 1.02 | -.06 | .07 | .01 | .01 | 5 | — | >.999 |
|  |  |  |  |  |  |  |  |  |  |  |  |  |
| Customer expertise → Cognitive CE | 16 | 3960 | .41* | 17% | 2.39 | .32 | .50 | .19 | .63 | 96* | 2395 | >.999 |
| Customer expertise → Emotional CE | 14 | 2615 | .18 | 3% | 1.44 | -.05 | .42 | -.38 | .74 | 397* | ‒ | >.999 |
| Customer expertise → Behavioral CE | 28 | 41640 | .26* | 7% | 1.70 | .22 | .30 | .11 | .41 | 420* | 11672 | >.999 |
| Customer expertise → Unidimensional CE | 5 | 1795 | .54* | 29% | 3.35 | .38 | .70 | .31 | .77 | 47* | 706 | >.999 |
| Customer expertise → Higher-order CE | 2 | 554 | .59* | 35% | 3.88 | .33 | .84 | .36 | .81 | 14* | 101 | >.999 |
|  |  |  |  |  |  |  |  |  |  |  |  |  |
| Education → Cognitive CE | 12 | 2704 | -.05 | 0% | .90 | -.10 | .00 | -.12 | .02 | 18 | ‒ | .830 |
| Education → Emotional CE | 12 | 2704 | -.06 | 0% | .89 | -.12 | .00 | -.16 | .05 | 25* | ‒ | .930 |
| Education → Behavioral CE | 17 | 4176 | -.04 | 0% | .92 | -.10 | .01 | -.14 | .05 | 35* | ‒ | .826 |
| Education → Unidimensional CE | 1 | 2399 | .09* | 1% | 1.20 | .09 | .09 | .09 | .09 | ‒ | ‒ | .997 |
| Education → Higher-order CE | ‒ | ‒ | ‒ |  |  | ‒ | ‒ | ‒ | ‒ | ‒ | ‒ | ‒ |
|  |  |  |  |  |  |  |  |  |  |  |  |  |
| Facilitating conditions → Cognitive CE | 29 | 7042 | .42* | 18% | 2.45 | .34 | .50 | .16 | .68 | 232* | 8117 | >.999 |
| Facilitating conditions → Emotional CE | 20 | 4766 | .53* | 28% | 3.26 | .45 | .61 | .31 | .75 | 112* | 5252 | >.999 |
| Facilitating conditions → Behavioral CE | 30 | 7791 | .50* | 25% | 3.00 | .42 | .58 | .23 | .77 | 280* | 12362 | >.999 |
| Facilitating conditions → Unidimen. CE | 10 | 3469 | .65* | 42% | 4.71 | .57 | .73 | .50 | .80 | 41* | 3919 | >.999 |
| Facilitating conditions → Higher-order CE | 6 | 1206 | .44* | 19% | 2.57 | .21 | .67 | .08 | .80 | 74* | 169 | >.999 |
|  |  |  |  |  |  |  |  |  |  |  |  |  |
| Firm competitiveness → Cognitive CE | 12 | 2097 | .34* | 12% | 2.03 | .27 | .41 | .21 | .47 | 26* | 734 | >.999 |
| Firm competitiveness → Emotional CE | 11 | 1200 | .45* | 20% | 2.64 | .39 | .51 | .39 | .51 | 10 | 779 | >.999 |
| Firm competitiveness → Behavioral CE | 20 | 8428 | .14* | 2% | 1.33 | .05 | .22 | -.11 | .38 | 238* | 1715 | >.999 |
| Firm competitiveness → Unidimensional CE | 3 | 1240 | .34* | 12% | 2.03 | .11 | .57 | .09 | .59 | 37* | 89 | >.999 |
| Firm competitiveness → Higher-order CE | 1 | 275 | .76* | 58% | 7.33 | .76 | .76 | .76 | .76 | — | — | >.999 |
|  |  |  |  |  |  |  |  |  |  |  |  |  |
| Functional value → Cognitive CE | 17 | 5641 | .47* | 22% | 2.77 | .39 | .56 | .26 | .69 | 129* | 5799 | >.999 |
| Functional value → Emotional CE | 19 | 6127 | .47* | 22% | 2.77 | .37 | .57 | .19 | .75 | 223* | 5996 | >.999 |
| Functional value → Behavioral CE | 44 | 18555 | .38* | 14% | 2.23 | .32 | .45 | .12 | .65 | 592* | 30157 | >.999 |
| Functional value → Unidimensional CE | 10 | 3987 | .54* | 29% | 3.35 | .46 | .63 | .37 | .71 | 57* | 3039 | >.999 |
| Functional value → Higher-order CE | 8 | 2919 | .43* | 18% | 2.51 | .29 | .56 | .19 | .66 | 76* | 1086 | >.999 |
|  |  |  |  |  |  |  |  |  |  |  |  |  |
| Gender (1 = female) → Cognitive CE | 12 | 2704 | -.02 | 0% | .96 | -.09 | .05 | -.14 | .10 | 29* | ‒ | .273 |
| Gender (1 = female) → Emotional CE | 12 | 2704 | -.11 | 1% | .80 | -.16 | -.06 | -.17 | -.06 | 16 | ‒ | >.999 |
| Gender (1 = female) → Behavioral CE | 21 | 7482 | .01 | 0% | 1.02 | -.04 | .05 | -.11 | .12 | 66* | ‒ | .218 |
| Gender (1 = female) → Unidimensional CE | 2 | 580 | -.05 | 0% | .90 | -.13 | .04 | -.05 | -.05 | ‒ | ‒ | .329 |
| Gender (1 = female) → Higher-order CE | ‒ | ‒ | ‒ | ‒ | ‒ | ‒ | ‒ | ‒ | ‒ | ‒ | ‒ | ‒ |
|  |  |  |  |  |  |  |  |  |  |  |  |  |
| Hedonic value → Cognitive CE | 8 | 1999 | .51* | 26% | 3.08 | .33 | .68 | .19 | .82 | 95* | 1083 | >.999 |
| Hedonic value → Emotional CE | 12 | 3503 | .69* | 48% | 5.45 | .63 | .76 | .56 | .83 | 32* | 4744 | >.999 |
| Hedonic value → Behavioral CE | 25 | 11722 | .43* | 18% | 2.51 | .34 | .51 | .16 | .70 | 401* | 13695 | >.999 |
| Hedonic value → Unidimensional CE | 4 | 1531 | .70* | 49% | 5.67 | .39 | 1.01 | .30 | 1.11 | 106* | 570 | >.999 |
| Hedonic value → Higher-order CE | 7 | 2555 | .50* | 25% | 3.00 | .25 | .75 | .07 | .92 | 213* | 1157 | >.999 |
|  |  |  |  |  |  |  |  |  |  |  |  |  |
| Social value → Cognitive CE | 14 | 5787 | .64* | 41% | 4.56 | .54 | .74 | .39 | .88 | 165* | 8216 | >.999 |
| Social value → Emotional CE | 15 | 6338 | .62* | 38% | 4.26 | .50 | .74 | .32 | .91 | 232* | 8164 | >.999 |
| Social value → Behavioral CE | 42 | 19968 | .49* | 24% | 2.92 | .43 | .55 | .24 | .74 | 581* | 48234 | >.999 |
| Social value → Unidimensional CE | 9 | 4068 | .68* | 46% | 5.25 | .60 | .77 | .52 | .85 | 51* | 4217 | >.999 |
| Social value → Higher-order CE | 10 | 4467 | .64* | 41% | 4.56 | .46 | .83 | .26 | 1.02 | 288* | 4109 | >.999 |

**Notes:** k = number of effect sizes, N = cumulative sample size, rwc = sample-weighted reliability adjusted average correlation, BESD = binomial effect size display, CI = 95% confidence interval, CR = 80% credibility interval, Q = Q statistic, FSN = fail-safe N, Power = results of power test. * *p* < .05.

**Web Appendix D: Method Appendix**

## Moderator Analysis

To assess the moderators and test the different engagement conceptualizations available in prior literature, we use a mixed-effects model. The collected effect sizes are nested within studies, so a traditional regression analysis may produce biased estimates (Bijmolt and Pieters 2001). Similar to Krasnikov and Jayachandran (2008) and Rosengren et al. (2020), we account for this possibility by using a mixed-effects multilevel model (Raudenbush and Bryk 2002), which includes fixed effects for the moderators, in addition to the random components. We calculate the multilevel models using hierarchical lineal modeling (HLM) software and specify the models as follows:

1. r_ij_ = γ_00_ + γ _01_ x (level 1-constructj) + γ_02_ x (level 1-constructj) + … + γ_10_ x (level 2-moderatorij) + γ_20_ x (level 2-moderatorij) + … + u_0j_ + e_ij_,

where r_ij_ is the ith effect size describing the relationship between engagement and the respective antecedent/consequence variable reported within the jth data set. With this approach, we conduct two analyses.

First, the multilevel approach has been used in meta-analysis to test moderators (Krasnikov and Jayachandran 2008). Thus, we calculate several multilevel models to assess the moderating effects of study characteristics on the relationships between CE and its antecedents and consequences. Similar to Rosengren et al. (2020), we calculate one model for each relationship. We assess the influence of the four substantive moderators characterizing the study context (e.g., platform type) and two method characteristics (e.g., research design). To assess the extent of multicollinearity among moderator variables, we calculate variance inflation factors. Then we complement these analyses with an additional subgroup analysis to facilitate the interpretation of the multilevel results (Hunter and Schmidt 2004). Although multilevel modeling is a superior analysis for moderator tests, because it considers nesting of the meta-analytic data and the simultaneous influence of all moderator variables, Grewal et al. (2018) suggest it should be complemented by a subgroup analysis to gain fuller insights.

Second, the multilevel approach appears in other meta-analyses that compare different construct conceptualizations. For example, LePine, Erez, and Johnson (2002) use it to compare the effect sizes of a second-order conceptualization of organizational citizenship behavior with a lower-order conceptualization. They also contrast the effect sizes of five lower-order organizational citizenship behavior dimensions with one another.

## Structural Equation Modeling

We tested the antecedents and consequences of CE using structural equation modeling, a testing approach that considers the interrelationships of the constructs simultaneously and provides fit statistics for model comparisons. In a comprehensive correlation matrix, we compile as many coded constructs as possible from our conceptual framework. Then the correlation matrix serves as input to LISREL 9.2, which we use to calculate the structural models. We rely on the harmonic mean across all sample sizes for this calculation, because it produces more conservative results than using the average sample size (Viswesvaran and Ones 1995). All constructs are measured with single indicators. We set the error variances of the constructs to 0, because the measurement already had been considered when we integrated the effect sizes.

**References**

Bijmolt, T. H., and Pieters, R. G. (2001), “Meta-analysis in marketing when studies contain multiple measurements,” *Marketing Letters*, *12*(2), 157-169.

Grewal, D., Puccinelli, N., and Monroe, K. B. (2018), “Meta-analysis: Integrating accumulated knowledge,” *Journal of the Academy of Marketing Science, 46*(1), 9-30.

Hunter, J. E., and Schmidt, F. L. (2004). *Methods of meta-analysis: Correcting error and bias in research findings*: Sage.

Krasnikov, A., and Jayachandran, S. (2008), “The relative impact of marketing, research-and-development, and operations capabilities on firm performance,” *Journal of Marketing*, *72*(4), 1-11.

LePine, J. A., Erez, A., and Johnson, D. E. (2002), “The nature and dimensionality of organizational citizenship behavior: A critical review and meta-analysis,” *Journal of Applied Psychology*, *87*(1), 52-65.

Raudenbush, S. W., and Bryk, A. S. (2002). *Hierarchical linear models: Applications and data analysis methods* Thousand Oaks: Sage.

Rosengren, S., Eisend, M., Koslow, S., and Dahlen, M. (2020), “A meta-analysis of when and how advertising creativity works,” *Journal of Marketing, 84*(6), 39-56.

Viswesvaran, C., and Ones, D. S. (1995), “Theory testing: Combining psychometric meta‐analysis and structural equations modeling,” *Personnel Psychology, 48*(4), 865-885.

**Web Appendix E: Measurement Based in Theory**

As displayed in Figure C.1, we identify five broad CE conceptualizations in prior literature, which can be classified into three groups, according to whether they consider CE a (1) dimensional construct (e.g., Calder et al. 2009), (2) multivariate construct (e.g., Hollebeek et al. 2014), or (3) unidimensional construct (e.g., Malthouse et al. 2016). Because we find no studies that use a profile or aggregate model, we do not discuss them further.

**Figure C.1. Structural Forms of CE**

**Notes**. CE = customer engagement, CCE = cognitive CE, ECE = emotional CE, and BCE = behavioral CE. We have tested the higher-order model and the unidimensional model 2 against the three CE dimensions using multilevel analysis. With structural equation modeling, we test whether the three CE dimensions are interrelated, as suggested in multivariate model 2, or unrelated, as suggested in multivariate model 1. The results support the proposed CE conceptualization.

## Multidimensional CE Construct

As displayed in Figure C.1, this conceptualization refers to a model in which the multidimensional construct exists at a different level than its dimensions (Law et al. 1998), which thus are manifestations of the construct. Accordingly, the engagement construct exists at a higher level, and CE dimensions are its manifestations. Engaged customers are eager to exhibit *simultaneous* cognitive, emotional, and behavioral engagement with the object (Clark et al. 2020). They are either high in all dimensions or low in all of them. This conceptualization reflects Kahn’s (1990) engagement theory, in which engagement occurs when people invest their “hands, head, and heart” in their work (Ashforth and Humphrey 1995, p. 110). Firms can actively influence employee engagement through organizational support and rewards (Rich et al. 2010); arguably, they should be able to exert similar influences on CE by developing marketing initiatives to support customers’ simultaneous behavioral, cognitive, and emotional investments in the firm (Beckers et al. 2018). In contrast, disengaged customers feature a simultaneous absence (withdrawal) of these investments (Maslowska et al. 2016). They do not want to think about the engagement object, experience related emotions, or engage in any behaviors to support the firm. Marketing strategies thus should display similar effects on the three CE dimensions, which are manifestations of the same underlying psychological state (Mowen and Voss 2008).

## Multivariate CE Construct

In this conceptualization, several lower-order constructs are conceptually related but distinguishable enough to be modeled separately (Edwards 2001), so a person’s investments in the interaction with the engagement object do not necessarily happen simultaneously or to the same extent (Rich et al. 2010). In Figure C.1, we depict two multivariate structural models. One approach asserts that the cognitive, emotional, and behavioral dimensions are distinguishable constructs, examined under the same engagement label. Engagement is not a construct but rather a useful label for a set of constructs that conceptually align (LePine et al. 2002). Despite its issues, this conceptualization is frequently used in marketing. A second approach presents CE dimensions as conceptually related but distinguishable enough to be measured and analyzed separately (Edwards 2001). Various attitudinal theories predict how cognitive and emotional constructs influence behavior (Fishbein and Ajzen 1975). Relative to CE, a multivariate structural model would predict that cognitive CE relates to emotional CE, which in turn influences behavioral CE. In detail, an engaged customer is eager to invest cognitively first, followed by emotional and behavioral investments (Oliver 1999). In testing this conceptualization, Hollebeek et al. (2014) find it is inferior to a multivariate model in which the engagement dimensions are unrelated. Both multivariate structural models predict that marketing strategies influence cognitive, emotional, and behavioral dimensions differently, because they represent separate constructs with unique variance (Edwards 2001).

## Unidimensional CE Construct

Figure C.1 reveals that many studies use unidimensional models. As detailed in our discussion of the behavioral CE school of thought (Harmeling et al. 2017), one model highlights how customers’ behavioral investments contribute, directly or indirectly, to firm-initiated activities (Pansari and Kumar 2017). Studies in this stream frequently refer to Pansari and Kumar’s (2017) engagement theory, which is based on the theory of reasoned action (Fishbein and Ajzen 1975) and a hierarchy of effects model of consumer behavior. Although a few scholars focus exclusively on emotional or cognitive CE dimensions, these approaches are less prominent, and their use is rather ad hoc (Brodie et al. 2011), so we do not discuss them in detail. Furthermore, multiple studies combine the three CE dimensions into one unidimensional notion. That is, they might mention the three engagement types but only use one set of cognitive, emotional, and behavioral engagement items, without differentiating among the dimensions. This approach is not guided by theory. Instead, scholars in pursuit of parsimony opt for it with the prediction that different engagement items correlate strongly, so combining them might make sense.

**References**

Ashforth, Blake E., and Ronald H. Humphrey (1995), “Emotion in the workplace: A reappraisal. *Human Relations*,” 48 (2), 97–125.

Beckers, Sander F.M., Jenny van Doorn, and Peter C. Verhoef (2018), “Good, better, engaged? The effect of company-initiated customer engagement behavior on shareholder value,” *Journal of the Academy of Marketing Science*, *46* (3), 366–83.

Brodie, R. J., Hollebeek, L. D., Jurić, B., and Ilić, A. (2011), “Customer engagement: Conceptual domain, fundamental propositions, and implications for research,” *Journal of Service Research*, *14*(3), 252-271.

Calder, Bobby J., Edward C. Malthouse, and Ute Schaedel (2009), “An experimental study of the relationship between online engagement and advertising effectiveness,” *Journal of Interactive Marketing, 23* (4), 321–31.

Clark, Moira K., Cristiana Raquel Lages, and Linda D. Hollebeek (2020), “Friend or foe? Customer engagement’s value-based effects on fellow customers and the firm,” *Journal of Business Research*, *121*, 549–56.

Edwards, Jeffrey R. (2001), “Multidimensional constructs in organizational behavior research,” *Organizational Research Methods*, *4* (2), 144–92.

Fishbein, Martin, and Icek Ajzen (1975), *Belief, attitude, intention, and behavior: An introduction to theory and research,* Reading: MA: Addison-Wesley.

Harmeling, C. M., Moffett, J. W., Arnold, M. J., and Carlson, B. D. (2017), “Toward a theory of customer engagement marketing,” *Journal of the Academy of Marketing Science*, 45(3), 312-335.

Hollebeek, Linda D., Mark S. Glynn, and Roderick J. Brodie (2014), “Consumer brand engagement in social media,” *Journal of Interactive Marketing, 28* (2), 149–65.

Kahn, William A. (1990), “Psychological conditions of personal engagement and disengagement at work,” *Academy of Management Journal*, *33* (4), 692–724.

Law, Kenneth S., Chi-Sum Wong, and William M. Mobley (1998), “Toward a taxonomy of multidimensional constructs,” *Academy of Management Review*, 23 (4), 741–55

LePine, Jeffrey A., Amir Erez, and Diane E. Johnson (2002), “The nature and dimensionality of organizational citizenship behavior,” *Journal of Applied Psychology*, *87* (1), 52–65.

Malthouse, E. C. et al. (2016), “Evidence that user-generated content that produces engagement increases purchase behaviours,” *Journal of Marketing Management, 32* (5-6), 427–44.

Maslowska, Ewa, Edward C. Malthouse, and Tom Collinger (2016), “The customer engagement ecosystem,” *Journal of Marketing Management, 32* (5-6), 469–501.

Mowen, John C., and Kevin E. Voss (2008), “On building better construct measures: Implications of a general hierarchical model,” *Psychology & Marketing*, *25* (6), 485–505.

Oliver, Richard L. (1999), “Whence consumer loyalty?” *Journal of Marketing, 63*(4), 33-44.

Pansari, A., and V. Kumar (2017), “Customer engagement: The construct, antecedents, and consequences,” *Journal of the Academy of Marketing Science, 45*(3), 294-311.

Rich, B., J. A. Lepine, and E. R. Crawford (2010), “Job engagement: Antecedents and effects on job performance,” *Academy of Management Journal*, *53* (3), 617–35.

**Web Appendix F:** **Testing Alternative CE Conceptualizations**

We assessed differences in effects sizes by CE conceptualization (Table 3 in manuscript). Regarding task-based initiatives, the effect of unidimensional CE (reliability-adjusted average correlation [rwc]= .54, *p* < .05) is greater than that of the higher-order conceptualization (rwc = .29, *p* < .05; Z_UCE-HCE_ = 8.60, *p* < .01). For experiential initiatives, the higher-order conceptualization offers effect sizes (rwc = .38, *p* < .05) that are like cognitive (Z_CCE-HCE_ = .03, *p >* .05) and emotional (Z_ECE-HCE_ = .15, *p* > .05) CE; those for the unidimensional conceptualization are greater (rwc = .50, *p* < .05; Z_UCE-HCE_ = 3.98, *p* < .01). Regarding product performance, the effect sizes for the unidimensional conceptualization are substantial (rwc = .50, *p* < .05), but those for the higher-order conceptualization are smaller (rwc = .23, *p* < .05; Z_UCE-HCE_ = 12.20, *p* < .01). For brand associations, the higher-order CE conceptualization (rwc = .65, *p* < .05) displays a stronger effect than the unidimensional version (rwc = .53, *p* < .05; Z_UCE-HCE_ = 8.79, *p* < .01). Regarding behavioral intentions and outcomes, the unidimensional (rwc = .58, rwc = .32, *p* < .05) and higher-order (rwc = .67, rwc = .27, *p* < .05) conceptualizations are all significant. The results point toward differences by CE dimension.

Next, we use multilevel modeling to estimate several models and compare alternative CE conceptualizations (LePine et al. 2002), using the criteria proposed by Mowen and Voss (2008), Edwards (2001), and LePine et al. (2002), namely, model fit, variance shared among the dimensions, and the relationships of the dimension of the construct with its antecedents and consequences. Model 1 includes the higher-order CE conceptualization and the three CE dimensions; Model 2 tests the unidimensional CE conceptualization against the three CE dimensions (effect sizes of customer CE and the four antecedents; effect sizes that link different antecedents are excluded); and Model 3 tests the three CE dimensions against one another. The correlations of the Level-1 and Level-2 variables are low (Table F.1), suggesting multicollinearity is low, in accordance with the low variance inflation factors we calculate (2.898 at Level 1 and 1.580 at Level 2).

**Table F.1. Correlations Among Moderator Variables**

**Panel A: Level 1 Moderators**

|  | **1.** | **2.** | **3.** | **4.** | **5.** | **6.** | **7.** | **8.** | **9.** |
| --- | --- | --- | --- | --- | --- | --- | --- | --- | --- |
| 1. Cognitive CE | 1.00 |  |  |  |  |  |  |  |  |
| 1. Emotional CE | -.24* | 1.00 |  |  |  |  |  |  |  |
| 1. Behavioral CE | -.44* | -.40* | 1.00 |  |  |  |  |  |  |
| 1. Unidimensional CE | -.18* | -.16* | -.29* | 1.00 |  |  |  |  |  |
| 1. Higher-order CE | -.16* | -.15* | -.26* | -.11* | 1.00 |  |  |  |  |
| 1. Task-based initiatives | -.04 | .00 | .05 | .01 | -.05 | 1.00 |  |  |  |
| 1. Experiential initiatives | -.04 | -.02 | .03 | -.03 | .06* | -.09* | 1.00 |  |  |
| 1. Product performance | .02 | -.01 | .00 | .00 | -.02 | -.13* | -.11* | 1.00 |  |
| 1. Brand associations | -.01 | -.04 | .00 | .03 | .03 | -.13* | -.11* | -.16* | 1.00 |

* *p* < .05.

Notes: Max. variance inflation factor = 2.898, excluding cognitive CE due to dummy coding of CE.

**Panel B: Level 2-Moderators**

|  | **1.** | **2.** | **3.** | **4.** | **5.** | **6.** | **7.** | **8.** | **9.** |
| --- | --- | --- | --- | --- | --- | --- | --- | --- | --- |
| 1. Platform type | 1.00 |  |  |  |  |  |  |  |  |
| 1. Initiator of interaction | -.11* | 1.00 |  |  |  |  |  |  |  |
| 1. Intensity of interaction | .24* | .16* | 1.00 |  |  |  |  |  |  |
| 1. Richness of interaction | .10 | .06 | .04 | 1.00 |  |  |  |  |  |
| 1. Single-industry | -.11* | .18* | -.09 | .11* | 1.00 |  |  |  |  |
| 1. Research design | -.08 | .07 | .10* | -.15* | -.05 | 1.00 |  |  |  |
| 1. Publication quality | -.01 | -.03 | -.11* | .04 | -.03 | -.06 | 1.00 |  |  |
| 1. Publication status | -.11* | .06 | .05 | .05 | -.13* | .02 | .54* | 1.00 |  |
| 1. Study year | -.02 | -.14* | -.07 | -.12* | -.15* | -.06 | .09 | .14* | 1.00 |

* *p* < .05.

Notes: Max. variance inflation factor = 1.554.

In Model 1 (Table F.2), at Level 1, we dummy-code the specific variable being measured with the effect size (i.e., CE type and antecedent). For example, we dummy-code whether the effect size represents a higher-order CE conceptualization or one of the three CE dimensions. At Level 2, we control for the influence of moderators. The nonsignificant estimate for the higher-order conceptualization (β = -.06, *p* = .11) indicates the same effect sizes as in the three CE dimensions. With Model 2 (Table F.2), we test the unidimensional CE conceptualization, which is not significant (β = .01, *p* = .70). However, we observe some differences across the three CE dimensions. In Model 3 (Table F.2), we test whether the dimensions display distinct effect sizes when tested against one another, at Level 1, with cognitive CE as the reference category. The results suggest again that emotional CE displays greater effect sizes than the other dimensions (β = .05, *p* < .01), whereas the effect sizes of behavioral CE are not significantly larger (β = -.02, *p =* .25). These findings affirm that scholars should differentiate the three CE types. The pseudo-R^2^ is highest for Model 1c.

**Table F.2. Testing Construct Conceptualizations across Antecedents using Multi-level Modeling**

|  | **Model 1a: Higher-Order CE vs. Three Dimensions** | | | **Model 1b: Unidimensional CE vs. Three Dimensions** | | | **Model 1c: Three CE Dimensions vs. One Another** | | |
| --- | --- | --- | --- | --- | --- | --- | --- | --- | --- |
| **Level 1: Effect Size Level** | **B** | **t-Ratio** | ***p*-value** | **B** | **t-Ratio** | ***p*-value** | **B** | **t-Ratio** | ***p*-value** |
| Intercept | .18 | 1.94 | .06 | .17 | 1.84 | .07 | .24 | 2.46 | .02 |
| Higher-order CE | -.06 | 1.60 | .11 | — |  |  | — |  |  |
| Unidimensional CE | — |  |  | .01 | .39 | .70 | — |  |  |
| Emotional CE | — |  |  | — |  |  | .05 | 2.65 | .01 |
| Behavioral CE | — |  |  | — |  |  | -.02 | 1.16 | .25 |
| Task-based initiatives | -.17 | 6.27 | <.001 | -.19 | 6.75 | <.001 | -.21 | 7.32 | <.001 |
| Experiential initiatives | -.10 | 2.97 | .00 | -.12 | 4.35 | <.001 | -.13 | 4.88 | <.001 |
| Brand associations | .08 | 3.21 | .00 | .03 | 1.99 | .05 | .04 | 2.39 | .02 |
|  |  |  |  |  |  |  |  |  |  |
| **Level 2: Study Level** |  |  |  |  |  |  |  |  |  |
| Platform type | .08 | 1.58 | .12 | .10 | 1.97 | .05 | .06 | 1.09 | .28 |
| Initiator of interaction | .01 | .12 | .90 | .00 | .04 | .97 | -.01 | .13 | .90 |
| Intensity of interaction | -.03 | .81 | .42 | -.01 | .30 | .76 | -.04 | 1.08 | .28 |
| Richness of interaction | -.01 | .28 | .78 | .00 | .06 | .95 | .01 | .27 | .79 |
| Single-industry | .01 | .34 | .73 | .03 | .95 | .34 | .02 | .43 | .67 |
| Research design | .21 | 3.46 | <.001 | .21 | 3.42 | <.001 | .20 | 3.38 | <.001 |
| Publication quality | -.02 | .94 | .35 | -.02 | 1.01 | .32 | -.02 | 1.16 | .25 |
| Publication status | .10 | 2.03 | .04 | .11 | 2.32 | .02 | .11 | 2.26 | .03 |
| Study year | .00 | .53 | .60 | .01 | 1.21 | .23 | .00 | .84 | .40 |
| Pseudo-R^2^ | 29% | | | 32% | | | 43% | | |

Notes: The three CE engagement dimensions are dummy-coded in Model 1c, and the marketing strategies are in Models 1a–1c. Cognitive CE and product performance are the reference categories in the respective models.

We complemented these models with additional analyses of differences across the three CE dimensions, according to the specific antecedent relationship (Table F.3). These models control for Level-2 influences. Again, the results reveal stronger effects of emotional CE for product performance (β = .08, *p* < .01) and brand associations (β = .10, *p <* .05). This further confirmation that scholars should use the three-dimensional CE conceptualization prompted us to continue our analyses with this conceptualization.

**Table F.2. Testing Three CE Dimensions by Antecedent, Multilevel Modeling**

| **Model** | **Predictor** | **B** | **t-ratio** | ***p*-value** |
| --- | --- | --- | --- | --- |
| Model 2a: Task-based initiative | Intercept | .12 | .94 | .35 |
|  | Emotional CE | .00 | .02 | .99 |
|  | Behavioral CE | -.04 | .97 | .34 |
|  | Platform type | -.10 | .90 | .38 |
|  | Initiator of interaction | .06 | .43 | .67 |
|  | Intensity of interaction | .02 | .33 | .74 |
|  | Richness of interaction | -.13 | 2.07 | .05 |
|  | Single-industry | -.01 | .11 | .92 |
|  | Research design | .30 | 3.27 | .00 |
|  | Publication quality | .05 | 1.65 | .11 |
|  | Publication status | .04 | .57 | .57 |
|  | Study year | -.01 | .96 | .34 |
| Model 2b: Exper. initiatives | Intercept | .26 | 1.11 | .28 |
|  | Emotional CE | -.01 | .31 | .76 |
|  | Behavioral CE | -.03 | .77 | .45 |
|  | Platform type | -.06 | .41 | .68 |
|  | Initiator of interaction | .38 | 1.71 | .10 |
|  | Intensity of interaction | -.30 | 3.23 | .00 |
|  | Richness of interaction | -.15 | 1.47 | .16 |
|  | Single-industry | -.04 | .54 | .59 |
|  | Research design | .21 | 2.14 | .04 |
|  | Publication quality | -.10 | 2.55 | .02 |
|  | Publication status | .38 | 3.80 | <.001 |
|  | Study year | -.02 | .99 | .33 |
| Model 2c: Product performance | Intercept | .31 | 2.63 | .01 |
|  | Emotional CE | .08 | 3.00 | .00 |
|  | Behavioral CE | -.01 | .32 | .75 |
|  | Platform type | .05 | .83 | .41 |
|  | Initiator of interaction | .00 | .01 | .99 |
|  | Intensity of interaction | -.06 | 1.16 | .25 |
|  | Richness of interaction | -.08 | 1.66 | .10 |
|  | Single-industry | .00 | .11 | .92 |
|  | Research design | .14 | 1.70 | .09 |
|  | Publication quality | -.02 | .85 | .40 |
|  | Publication status | .03 | .42 | .68 |
|  | Study year | -.01 | 1.33 | .19 |
| Model 2d: Brand associations | Intercept | .14 | .84 | .40 |
|  | Emotional CE | .10 | 2.18 | .03 |
|  | Behavioral CE | -.01 | .34 | .74 |
|  | Platform type | .06 | .78 | .44 |
|  | Initiator of interaction | -.03 | .51 | .61 |
|  | Intensity of interaction | .01 | .11 | .91 |
|  | Richness of interaction | .15 | 1.64 | .11 |
|  | Single-industry | .02 | .36 | .72 |
|  | Research design | .30 | 2.16 | .04 |
|  | Publication quality | -.03 | .97 | .34 |
|  | Publication status | .05 | .63 | .53 |
|  | Study year | .00 | .43 | .67 |

Notes: The three CE engagement dimensions are dummy-coded in Models 2a–d. Cognitive CE is the reference category.

**References**

Edwards, Jeffrey R. (2001), “Multidimensional constructs in organizational behavior research,” *Organizational Research Methods*, *4* (2), 144–92.

LePine, Jeffrey A., Amir Erez, and Diane E. Johnson (2002), “The nature and dimensionality of organizational citizenship behavior,” *Journal of Applied Psychology*, *87* (1), 52–65.

Mowen, John C., and Kevin E. Voss (2008), “On building better construct measures: Implications of a general hierarchical model,” *Psychology & Marketing*, *25* (6), 485–505.

**Web Appendix G: Correlation Matrix**

|  | **1.** | **2.** | **3.** | **4.** | **5.** | **6.** | **7.** | **8.** | **9.** |
| --- | --- | --- | --- | --- | --- | --- | --- | --- | --- |
| 1. Cognitive CE |  | 86 | 115 | 15 | 11 | 33 | 31 | 57 | 16 |
| 1. Emotional CE | .69 |  | 99 | 18 | 11 | 25 | 23 | 54 | 14 |
| 1. Behavioral CE | .54 | .57 |  | 48 | 33 | 60 | 63 | 113 | 44 |
| 1. Task-based initiatives | .37 | .48 | .21 |  | 20 | 18 | 13 | 21 | 16 |
| 1. Experiential initiatives | .38 | .38 | .12 | .05 |  | 15 | 14 | 16 | 12 |
| 1. Product performance | .50 | .60 | .47 | .42 | .46 |  | 21 | 36 | 15 |
| 1. Brand associations | .58 | .59 | .41 | .34 | .27 | .54 |  | 41 | 15 |
| 1. Behavioral intentions | .50 | .55 | .49 | .43 | .34 | .52 | .54 |  | 19 |
| 1. Behavioral outcome | .21 | .28 | .24 | .13 | .16 | .24 | .42 | .41 |  |

Notes: The lower half of the table displays the sample-size weighted, reliability-adjusted correlations; the upper half displays the number of effect sizes. Harmonic mean of all sample sizes: 2,947.

**Web Appendix H: Testing Indirect Effects of Antecedents through CE Dimensions**

| **Relationship** | **B** | **t-value** |
| --- | --- | --- |
| **ANTECEDENTS** |  |  |
| ***CE Marketing*** |  |  |
| Task-based initiatives → Cognitive CE | — |  |
| Task-based initiatives → Emotional CE | .07* | 9.90 |
| Task-based initiatives → Behavioral CE | .12* | 13.98 |
| Experiential initiatives → Cognitive CE | — |  |
| Experiential initiatives → Emotional CE | .08* | 11.39 |
| Experiential initiatives → Behavioral CE | .10* | 12.57 |
| ***Traditional Marketing*** |  |  |
| Product performance → Cognitive CE | — |  |
| Product performance → Emotional CE | .04* | 5.52 |
| Product performance → Behavioral CE | .10* | 9.98 |
| Brand associations → Cognitive CE | — |  |
| Brand associations → Emotional CE | .16* | 16.97 |
| Brand associations → Behavioral CE | .20* | 18.63 |
| **CE DIMENSIONS** |  |  |
| Cognitive CE → Emotional CE | — |  |
| Cognitive CE → Behavioral CE | .12* | 11.55 |
| Emotional CE → Behavioral CE | — |  |
| **CONSEQUENCES** |  |  |
| Task-based initiatives → Behavioral intentions | .04* | 10.96 |
| Experiential initiatives → Behavioral intentions | .03* | 10.24 |
| Product performance → Behavioral intentions | .08* | 10.93 |
| Brand associations → Behavioral intentions | .06* | 12.82 |
| Cognitive CE → Behavioral intentions | .11* | 13.32 |
| Emotional CE → Behavioral intentions | .08* | 10.50 |
| Behavioral CE → Behavioral intentions | — |  |
| Task-based initiatives → Behavioral outcome | .07* | 10.71 |
| Experiential initiatives → Behavioral outcome | .06* | 9.78 |
| Product performance → Behavioral outcome | .03* | 5.71 |
| Brand associations → Behavioral outcome | .08* | 10.80 |
| Cognitive CE → Behavioral outcome | .03* | 9.39 |
| Emotional CE → Behavioral outcome | .02* | 8.23 |
| Behavioral CE → Behavioral outcome | .07* | 10.60 |
| Behavioral intentions → Behavioral outcome | — |  |
|  |  |  |
| Model fit: |  |  |
| Goodness-of-fit index | .97 |  |
| Root mean residual | .04 |  |
| Standardized mean residual | .04 |  |

* *p* < .05.

**Web Appendix I:** **Testing Reverse Causality of CE Dimensions in Meta-Analytic Framework**

| **Relationship** | **Estimate** | **t-value** |
| --- | --- | --- |
| **ANTECEDENTS** |  |  |
| ***CE Marketing*** |  |  |
| Task-based initiatives → Cognitive CE | .18* | 12.78 |
| Task-based initiatives → Emotional CE | .22* | 16.84 |
| Task-based initiatives → Behavioral CE | — |  |
| Experiential initiatives → Cognitive CE | .25* | 17.85 |
| Experiential initiatives → Emotional CE | .14* | 10.61 |
| Experiential initiatives → Behavioral CE | — |  |
| ***Traditional Marketing*** |  |  |
| Product performance → Cognitive CE | — |  |
| Product performance → Emotional CE | .10* | 6.19 |
| Product performance → Behavioral CE | .35* | 17.95 |
| Brand associations → Cognitive CE | .31* | 20.30 |
| Brand associations → Emotional CE | .16* | 10.78 |
| Brand associations → Behavioral CE | .22* | 11.28 |
| **CE DIMENSIONS** |  |  |
| Behavioral CE → Cognitive CE | .35* | 23.68 |
| Behavioral CE → Emotional CE | .24* | 16.95 |
| Cognitive CE → Emotional CE | .28* | 16.93 |
| **CONSEQUENCES** |  |  |
| Task-based initiatives → Behavioral intentions | .19* | 11.17 |
| Experiential initiatives → Behavioral intentions | .12* | 7.11 |
| Product performance → Behavioral intentions | .13* | 6.45 |
| Brand associations → Behavioral intentions | .25* | 12.83 |
| Cognitive CE → Behavioral intentions | .09* | 4.08 |
| Emotional CE → Behavioral intentions | .13* | 5.65 |
| Task-based initiatives → Behavioral outcome | — |  |
| Experiential initiatives → Behavioral outcome | — |  |
| Product performance → Behavioral outcome | — |  |
| Brand associations → Behavioral outcome | .28* | 14.05 |
| Behavioral CE → Behavioral outcome | — |  |
| Cognitive CE → Behavioral outcome | — |  |
| Emotional CE → Behavioral outcome | — |  |
| Behavioral intentions → Behavioral outcome | .26* | 12.99 |

* *p* < .05.

Notes: When we compare the proposed model with this model, we note that they differ in their complexity (i.e., number of relationships). Thus, we use fit criteria that consider differences in model complexity. Both the parsimonious normed fit index (PNFI) and parsimony goodness-of-fit index (PGFI) suggest the proposed model outperforms the alternative model (PNFI_proposed_ = .30, PNFI_alternative_ = .27; PGFI_proposed_ = .24, PGFI_alternative_ = .22).

| **Web Appendix J: Results of Subgroup Analysis** | | | | | | | | | | | |
| --- | --- | --- | --- | --- | --- | --- | --- | --- | --- | --- | --- |
| **Relationship** | **k** |  | **High: Contin. inter. ^a^  Low: Spot interaction** | **High: Rich interact. ^a^ Low: Lean interact.** | **High: Virt. platform ^a^  Low: Phys. platform** | **High: Customer-init. ^a^  Low: Firm-initiated** | **High: Single-industry ^a^ Low: Multiple indust.** | **High: Survey ^a^ Low: Other design** | **Publication quality ^b^** | **High: Published ^a^**  **Low: Not published** | **Study year ^b^** |
| Task-based initiatives → Cognitive CE | 15 | r_high_ | .51 ^A^ | .08 ^A^ | — | — | .28 ^A^ | — | .09 | .42 ^A^ | .10 |
|  |  | r_low_ | .30 | .42 | — | — | .49 | — |  | .25 |  |
| Task-based initiatives → Emotional CE | 18 | r_high_ | .54 ^A^ | .29 ^A^ | — | — | .43 ^A^ | — | .53 ^A^ | .57 ^A^ | -.37 |
|  |  | r_low_ | .42 | .49 | — | — | .58 | — |  | .37 |  |
| Task-based initiatives → Behavioral CE | 48 | r_high_ | .25 ^A^ | .18 | .20 ^A^ | .55 ^A^ | .21 | .37 ^A^ | .27 ^A^ | .21 ^A^ | .08 |
|  |  | r_low_ | .18 | .21 | .49 | .19 | .20 | .02 |  | .19 |  |
| Experiential initiatives → Cognitive CE | 11 | r_high_ | .38 | .39 | — | .39 | .37 | .37 | .00 | .38 | .00 |
|  |  | r_low_ | .38 | .37 | — | .37 | .38 | .39 |  | .37 |  |
| Experiential initiatives → Emotional CE | 11 | r_high_ | .43 | .45 ^A^ | — | .45 | .34 | .35 | .27 | .44 ^A^ | -.32 |
|  |  | r_low_ | .35 | .34 | — | .35 | .44 | .45 |  | .33 |  |
| Experiential initiatives → Behavioral CE | 33 | r_high_ | .10 | .08 ^A^ | .10 ^A^ | .34 ^A^ | .08 ^A^ | .26 ^A^ | -.08 | .13 ^A^ | .01 |
|  |  | r_low_ | .12 | .13 | .30 | .11 | .18 | .00 |  | .07 |  |
| Product performance → Cognitive CE | 33 | r_high_ | .40 ^A^ | .49 | .48 ^A^ | .54 | .52 ^A^ | .50 | .06 | .51 | .05 |
|  |  | r_low_ | .53 | .50 | .61 | .49 | .46 | .49 |  | .48 |  |
| Product performance → Emotional CE | 25 | r_high_ | .60 | .66 | .62 | .55 ^A^ | .61 | — | -.31 | .61 | -.04 |
|  |  | r_low_ | .61 | .61 | .58 | .62 | .60 | — |  | .60 |  |
| Product performance → Behavioral CE | 60 | r_high_ | .49 ^A^ | .38 ^A^ | .47 | .51 ^A^ | .45 ^A^ | .48 ^A^ | -.10 | .47 | -.09 |
|  |  | r_low_ | .46 | .48 | .47 | .46 | .50 | .14 |  | .45 |  |
| Brand associations → Cognitive CE | 31 | r_high_ | .57 | .72 ^A^ | .59 | .62 ^A^ | .57 ^A^ | .58 | .11 | .60 ^A^ | -.16 |
|  |  | r_low_ | .60 | .55 | .57 | .57 | .61 | .65 |  | .52 |  |
| Brand associations → Emotional CE | 23 | r_high_ | .52 ^A^ | .54 | .58 | .70 ^A^ | .59 | .59 | -.17 | .58 | .29 |
|  |  | r_low_ | .66 | .59 | .62 | .56 | .59 | .59 |  | .61 |  |
| Brand associations → Behavioral CE | 63 | r_high_ | .50 ^A^ | .41 | .40 ^A^ | .52 ^A^ | .43 ^A^ | .44 ^A^ | -.23 ^A^ | .39 ^A^ | .11 |
|  |  | r_low_ | .33 | .41 | .44 | .39 | .38 | .17 |  | .46 |  |
| Cognitive CE → Behavioral intentions | 56 | r_high_ | .49 | .53 | .48 ^A^ | .50 | .47 ^A^ | .49 ^A^ | .26 ^A^ | .51 ^A^ | -.25 ^A^ |
|  |  | r_low_ | .51 | .50 | .62 | .50 | .54 | .57 |  | .47 |  |
| Emotional CE → Behavioral intentions | 54 | r_high_ | .56 | .62 | .54 ^A^ | .66 ^A^ | .56 | .55 ^A^ | .07 | .54 ^A^ | -.02 |
|  |  | r_low_ | .55 | .56 | .61 | .47 | .56 | .59 |  | .60 |  |
| Behavioral CE → Behavioral intentions | 113 | r_high_ | .50 ^A^ | .55 ^A^ | .46^A^ | .54^A^ | .47 ^A^ | .48 ^A^ | -.18 ^A^ | .48 ^A^ | .07 |
|  |  | r_low_ | .47 | .48 | .60 | .46 | .52 | .67 |  | .51 |  |
| Cognitive CE → Behavioral outcome | 16 | r_high_ | .15 ^A^ | .36 ^A^ | .21 | .23 | .22 ^A^ | .28 ^A^ | -.14 | .18 ^A^ | .25 |
|  |  | r_low_ | .29 | .20 | .23 | .21 | .12 | .13 |  | .31 |  |
| Emotional CE → Behavioral outcome | 14 | r_high_ | .22 ^A^ | .28 | — | — | .30 ^A^ | .38 ^A^ | -.56 ^A^ | .19 ^A^ | .55 ^A^ |
|  |  | r_low_ | .44 | .39 | — | — | .14 | .16 |  | .38 |  |
| Behavioral CE → Behavioral outcome | 44 | r_high_ | .21 ^A^ | .20 ^A^ | .22 ^A^ | .37 ^A^ | .25 ^A^ | .29 ^A^ | -.08 | .29 ^A^ | .15 |
|  |  | r_low_ | .28 | .28 | .34 | .23 | .21 | .16 |  | .15 |  |

Notes: The estimates displayed in the table are (a) reliability-adjusted and weighted correlations for the dichotomous moderators, and (b) correlations between effect size and moderator variables for the continuous moderators (Hunter and Schmidt 2004). The superscript (A) indicates significant moderating effects.

**Web Appendix K: Avenues for Research on CE Construct**

Scholars should gather insights from consumer psychology and marketing research to extend our CE marketing framework. For example, we conceptualize CE as a multivariate construct with three interrelated dimensions, in line with established attitudinal theories (Fishbein and Ajzen 1975). However, considerations of emotion–cognition interactions could provide even more nuanced views on CE dimensions (Pessoa 2018), by addressing how processes that are generally considered cognitive might be altered by emotions, or vice versa.

First, emotions are associated with cognitive appraisal processes—conscious or unconscious evaluations of events or thoughts (Bagozzi et al. 1999; Pessoa 2008). Research on emotion regulation describes processes by which people stifle laughter when observing another’s misfortune or appear enthusiastic about a business opportunity (Webb et al. 2012). Several frameworks detail how people regulate their emotions, but the process model of emotion regulation is widely used (Gross 1998), with its four underlying processes (i.e., situation selection, situation modification, attentional deployment, and cognitive change). This stream of research provides CE scholars with some interesting constructs and processes. For example, they might determine when and why cognitively demanding activities (i.e., cocreation tasks) appear stressful (McRae and Gross 2020). Then they could explain better when positive (i.e., thrilled or enthused by a demanding situation) versus negative (i.e., being frustrated or exhausted) emotional CE is likely to develop (Califf et al. 2020).

Second, emotions can drive cognitive processes (Bagozzi et al. 1999) and influence memory effects, including information encoding, retrieval (Bagozzi et al. 1999), and processing (Schwarz 2011). Being in a positive or negative emotional state influences how people recall information about an event or attend to a given environment (Gable and Harmon-Jones 2008). This literature stream also provides insights into emotion–cognition interplays, novel processes, and theories (Schwarz 2011). For example, CE research could consider if the congruence between induced emotions and an engagement situation eases cognitive information processing (i.e., increases attention) (Schwarz et al. 2021).

Third, as discussions in consumer psychology and neuroscience indicate, the establishment of psychological engagement dimensions may go beyond a dual process approach (Ochsner and Phelps 2007). Studies should address physiological aspects of emotions (e.g., bodily responses, such as increased heart rate) and psychological aspects (e.g., feeling of happiness), the timing of emotions and cognitions, and the duration of emotions (Dolcos et al. 2020). For example, CE scholars could try to disentangle the duration of emotional engagement (short- vs. long-term enthusiasm) and whether it relates differentially to behavioral CE over time.

**References**Bagozzi, R. P., M. Gopinath, and P. U. Nyer (1999), “The role of emotions in marketing,” *Journal of the Academy of Marketing Science,* 27(2), 184-206.

Califf, C.B., S. Sarker, and S. Sarker (2020), “The bright and dark sides of technostress: A mixed-methods study involving healthcare IT,” *MIS Quarterly*, 44(2), 809–56.

Dolcos, F. et al. (2020), “Neural correlates of emotion-attention interactions: From perception, learning, and memory to social cognition, individual differences, and training interventions,” *Neuroscience & Biobehavioral Reviews*, 108, 559-601.

Fishbein, Martin, and Icek Ajzen (1975), *Belief, attitude, intention, and behavior: An introduction to theory and research,* Reading: MA: Addison-Wesley.

Gable, P.A., and E. Harmon-Jones (2008), “Approach-motivated positive affect reduces breadth of attention,” *Psychological Science*, 19(5), 476-482.

Gross, J.J. (1998), “Antecedent-and response-focused emotion regulation: divergent consequences for experience, expression, and physiology,” *Journal of Personality and Social Psychology*, 74(1), 224–237.

McRae, K., and J.J. Gross (2020), “Emotion regulation,” *Emotion*, 20(1), 1-9.

Ochsner, K. N., and E. Phelps (2007), “Emerging perspectives on emotion–cognition interactions,” *Trends in Cognitive Sciences*, 11(8), 317–318.

Pessoa, L. (2008), “On the relationship between emotion and cognition,” *Nature Reviews Neuroscience*, 9(2), 148–58.

____ (2018), “Understanding emotion with brain networks,” *Current Opinion in Behavioral Sciences,* 19, 19-25.

Schwarz, N. (2011), “Feelings-as-information theory,” *Handbook of Theories of Social Psychology*, 1, 289–308.

____, M. et al. (2021), “Metacognitive experiences as information,” *Consumer Psychology Review*, 4(1), 4–25.

Webb, T.L., E. Miles, and P. Sheeran (2012), “Dealing with feeling: a meta-analysis of the effectiveness of strategies derived from the process model of emotion regulation,” *Psychological Bulletin*, 138(4), 775–808.
